# Enrichment of sweat-derived extracellular vesicles of human and bacterial origin for biomarker identification

**DOI:** 10.7150/ntno.87822

**Published:** 2024-01-01

**Authors:** Artem Zhyvolozhnyi, Anatoliy Samoylenko, Geneviève Bart, Anna Kaisanlahti, Jenni Hekkala, Olha Makieieva, Feby Pratiwi, Ilkka Miinalainen, Mika Kaakinen, Ulrich Bergman, Prateek Singh, Tuomas Nurmi, Elham Khosrowbadi, Eslam Abdelrady, Sakari Kellokumpu, Susanna Kosamo, Justus Reunanen, Juha Röning, Jussi Hiltunen, Seppo J. Vainio

**Affiliations:** 1 Faculty of Biochemistry and Molecular Medicine, Biocenter Oulu, InfoTech Oulu, University of Oulu, Oulu, Finland.; 2 Faculty of Medicine, Biocenter of Oulu, University of Oulu, Finland.; 3 Department of Computer Science and Engineering, University of Oulu, Finland.; 4 VTT-Technical Research Centre of Finland, Oulu, Finland.

**Keywords:** extracellular vesicles, sweat, proteomics, alginate, bacteria

## Abstract

Sweat contains biomarkers for real-time non-invasive health monitoring, but only a few relevant analytes are currently used in clinical practice. In the present study, we investigated whether sweat-derived extracellular vesicles (EVs) can be used as a source of potential protein biomarkers of human and bacterial origin.

**Methods:** By using ExoView platform, electron microscopy, nanoparticle tracking analysis and Western blotting we characterized EVs in the sweat of eight volunteers performing rigorous exercise. We compared the presence of EV markers as well as general protein composition of total sweat, EV-enriched sweat and sweat samples collected in alginate skin patches.

**Results:** We identified 1209 unique human proteins in EV-enriched sweat, of which approximately 20% were present in every individual sample investigated. Sweat derived EVs shared 846 human proteins (70%) with total sweat, while 368 proteins (30%) were captured by medical grade alginate skin patch and such EVs contained the typical exosome marker CD63. The majority of identified proteins are known to be carried by EVs found in other biofluids, mostly urine. Besides human proteins, EV-enriched sweat samples contained 1594 proteins of bacterial origin. Bacterial protein profiles in EV-enriched sweat were characterized by high interindividual variability, that reflected differences in total sweat composition. Alginate-based sweat patch accumulated only 5% proteins of bacterial origin.

**Conclusion:** We showed that sweat-derived EVs provide a rich source of potential biomarkers of human and bacterial origin. Use of commercially available alginate skin patches selectively enrich for human derived material with very little microbial material collected.

## Introduction

Despite the growing need to develop P4 (predictive, preventive, personalized and participatory) medicine, non-invasive, real-time health monitoring is still limited to only few parameters. One of the most promising body fluids to exploit in this context is sweat, as it may provide an alternative to blood biomarker analysis since it is collected non-invasively and in real-time.

Sweat has already attracted research interest for diagnostic purposes 1,2 in mental illness 3, tuberculosis 4, Behcet's disease 5 and diabetes 6,7. However, at present it is only routinely used in diagnosis of cystic fibrosis in newborn babies 8 due to limited number of known analytes 2,9-11. The discovery that biofluids transport secreted extracellular vesicles (EVs) carrying a wealth of analytes, including proteins, lipids, metabolites and many RNA species, means that it may be possible to exploit EVs to extract information even from a harsh environment like the skin surface 12,13. Bacterial EVs 14 derived from skin microbiota can also be a valuable source of health-related analytes 15,16. However, the lack of methods to separate bacterial and human-derived components of sweat presents a challenge in using sweat for health monitoring 17. An additional hurdle is the variation in amount of sweat collected and concentrations of analytes 10,11. This problem may be solved with the development of new wearable sensors 2,18 with detection capabilities for sweat glucose 19,20, alcohol 19, cortisol 21, lactate 22 alone or in combinations. In these wearable devices, analytes are concentrated using various strategies making the amount of liquid sweated less of a drawback. What would be the best way to design a wearable device for collection of sweat EVs is not known. One option may be using of alginate-based hydrogels that are biocompatible, biodegradable, inexpensive 20 and suitable for encapsulation of exosomes 23.

Microfluidic platforms for blood EV analysis to detect specific mRNA 24, miRNA sensors for EV detection, as well as microarray chips for detecting EV protein biomarkers in blood 25,26 have already been developed. Wearable sensors to monitor proteins and metabolites in “alternative” body fluids such as sweat, saliva, and interstitial fluid are also being developed 18. To apply these innovations to sweat EVs analysis we need a better characterization of their cargo, such as RNA and proteins. RNA and DNA composition of EV-enriched human sweat 27,28, metabolomics analysis of sweat 11,29-32 and sweat EVs 33, as well as proteomics analysis of whole sweat 3,4,9,11,34-36 and sweat-derived exosomes of human origin pooled from several individuals 37 have been reported. Our aim in the present study was to characterize protein cargo of EVs in individual unpooled sweat samples collected during a biking exercise. We report here that human EV-enriched sweat contains proteins originating both form host and microbiome and their composition reflects protein profiles of total sweat. We also applied an alginate skin patch for sweat collection and demonstrated that EV proteins can be analyzed from such patches. These skin patches selectively enriched human proteins while only few bacterial proteins could be identified. Taken together, these data show that the development of wearable devices for real-time analysis of biomarkers in sweat EVs can be achieved using material already being developed for detection of other parameter of sweat 38-40.

## Methods

### Sweat collection and exercise protocol

Eight healthy male volunteers (age 35 ± 10 years) were given information about the study and provided limited health and fitness self-assessment and informed consent. Ethical permission (EETTMK:110/2015) was approved by ethical committee of Oulu University medical School according to the Finnish Medical Research Act (488/1999). Volunteers were asked to avoid using soap and perfume for 24 hours before the exercise and to shower with water only for 15 minutes immediately before exercise. Volunteers used bike (ProSpinner spinning bike, Karhu) at room temperature (25 ^o^C) for 30 minutes including 10 minutes warm up; pace and effort was up to the individual. Sweat was collected from the upper body, arms and torso as described 27 by using one size polyethylene raincoat (Tammer Brands Oy, Finland) and disposable gloves (024199, GIA, France). After exercise sweat was taken by cutting the gloves at finger level and inserting sterile pipet to aspirate fluid. When there was sweat accumulated in the coat front, it was also collected in the same manner. Head dripping sweat was collected in the raincoat hood.

Sweat samples (31 ± 26 ml) were passed through a 40 μm cell strainer (22363547, Fisherbrand), then through a 0.8 μm filter (Millipore). Filtered sweat was concentrated on Centricon Plus-70 centrifugal filter, cut-off 100 kDa (UFC710008, Millipore, Ireland) according to manufacturer instructions. One half of the concentrated sweat was kept at -70 ^o^C for further analysis (“total sweat”), and another half was used for EV isolation.

### EV samples preparation

EV samples were prepared separately from each individual collection using ExoEasy kit (76064, Qiagen, Germany) as per manufacturer's protocol. Briefly, one volume of buffer XBP was added to 1 volume of sample and mixed well by inverting. The sample/XBP mix was centrifuged at 500 x g for 1 min. The flow-through was discarded, and 10 mL of buffer XWP was added and centrifuged at 4000 x g for 5 min to remove the residual buffer from the column. The spin column was transferred to a fresh collection tube. Buffer XE was added to the membrane (250-400 µL) and incubated for 1 min. The eluate was collected by centrifuging at 500 x g for 5 min.

### Skin patches

The skin patch consisted of fixation film (1626W, 3M Tegaderm Film, Germany) and Calcium alginate dressing (Cutiderm, JFA Medical, UK). The 25 cm^2^ patch was placed approximately 10 cm below the armpit before biking exercise (Supplementary Figure 1). After exercise, dressing was collected in a 50 ml tube (62.547.254 Rohre, Germany) containing 30 ml of 1xPBS (20-031-CV, Corning, USA) and vortexed vigorously. The sample was passed through a 40 μm cell strainer (22363547, Fisherbrand) and centrifugated (4000 x g 15 min) to remove particles originating from skin dressing. Debris-free supernatant was concentrated on Centricon Plus-70 centrifugal filter. The concentrated skin patch sweat was additionally centrifuged (21000 x g, 15 min), and the supernatant was collected.

### ExoView analysis

For analyzing expression of EV biomarkers and EV quantification the ExoView R100 platform (NanoView Biosciences, Boston) was used. Human ExoView Tetraspanin (EV-TETRA-C) kit was used for the analysis. The samples (EV, total sweat and patch) were processed according to the manufacturer's protocol. 1 µg of protein samples were carefully loaded onto each chip and incubated for 24 h. After that, the chips were washed three times on an orbital shaker to remove unbound particles. The chips were incubated for one hour with the human anti-CD81 (BD Pharmingen 555675), anti-CD63 (BD Pharmingen 556019), and anti-CD9 (Biolegend V P018) fluorescently labelled antibodies. Mouse IgG (Biolegend 400101) were used as controls. The immunostained chips were washed three times in PBS, once in deionized water and dried. Imaging and data acquisition of the stained chips were performed with the ExoView R100 (NanoView Biosciences) and the data analysis with the ExoViewer 3 (NanoView Biosciences).

### Western blotting

Proteins (10 µg for total sweat and 2 µg for EV-enriched sample) were separated on 10% SDS PAA gel, then transferred to nitrocellulose membrane. Anti-CD63 (Santa Cruz, sc-365604) dilution 1:1000 was used for detection. Total proteins on membranes were stained with total stain Q (Azure Biosystems). The respective secondary peroxidase-conjugated IgG antibodies (Invitrogen) at 1:5000 dilutions were then applied to the membranes. The Lumi-Light Western Blotting Substrate (Roche Diagnostics, Switzerland) was used to visualize the bound antibodies.

### Electron microscopy and immunoelectron microscopy

EV samples were analyzed by transmission electron microscopy (TEM). 2 µl of each sample were deposited on a Formvar carbonated grid (glow-discharged) and after negative staining with 2% uranyl acetate and immunostaining with anti-CD63 antibody (Abcam Ab193349; 1:50 dilution) examined using the Tecnai G2 Spirit transmission electron microscope (FEI, Eindhoven, The Netherlands). Protein A-gold complex (10 nm) served to detect the primary anti-CD63 antibody. Images were captured with a charge-coupled device camera (Quemesa, Olympus Soft Imaging Solutions GMBH, Münster, Germany) at 1:49,000, 1:30,000, and 1:18,500 magnifications.

### Nanoparticle tracking analysis

Nanoparticle tracking analysis (NTA) was performed using a Nano Sight NS300 (Malvern Panalytical) equipped with a 405 nm laser. Temperature was monitored throughout the measurements. Four or eight 60 s videos were recorded of each sample with camera level 14 and detection threshold set up at 3. Data were analyzed with NTA software version 3.4. Double distilled water was used to dilute the starting material.

### Lectin microarray analyses

Sweat samples were first centrifuged at 12,000×*g* at 4 °C for 15 min, after which 12 μg of total protein was labeled with 6 μg of NHS activated DyLight 633 protein dye (Thermo Scientific, Waltham, USA) in 50 μL of labeling buffer for 1h at RT with constant agitation (600 rpm). The reaction was quenched at RT (1 hr) by adding 50 mM ethanolamine in Tris-HCl/150 mM NaCl buffer in the labeling mix. Labeled samples were then cleared by centrifugation (12,000×*g* for 10 min in RT) and applied onto pre-printed and pre-quenched (with 50 mM ethanolamine) Nexterion H microarray slides (Schott, Germany), and further incubated in a humidified chamber with constant agitation for 2 h in RT. Slides were then washed 5 times for 5 min each with the assay buffer. Array images were generated using the Genepix 4200AL laser scanner (Auto Loader, Axon Instruments) using an appropriate filter set for the DyLight 633™ dye. The mean intensities of bound label were quantified in triplicate from 4 parallel arrays (36 measurement spots/sample) using the GenePix® Pro Microarray Analysis Software. The lectins used for microarray printing were purchased from the Vector Laboratories (Youngstown, OH, USA). Their sugar specificities are shown as described in the manufacturer's product sheets (Supplementary Table 7).

### Proteomics analysis

Proteins were extracted from individual EV samples by methanol/chloroform precipitation. Dried protein pellets were diluted in 4× Laemmli buffer containing 10% β-mercaptoethanol, loaded into 12% SDS polyacrylamide (PAA) gel (12% Mini-PROTEAN TGX Precast Protein Gel, Bio-Rad) and run for maximum 15 min at 100-110 V. SDS gel pieces stained with Sypro Ruby (Sigma, S4942) were cut out and processed as follows: 3 × 5 min washing steps with 50 mM ammonium-bicarbonate in 40% acetonitrile/60% water to destain the gel, reduction with 20 mM dithiothreitol for 30 min at room temperature, alkylation with 45 mM iodoacetamide for 30 min at room temperature, washing and tryptic digestion with 5 μl of trypsin solution (20 ng/μl proteomics grade trypsin (Sigma) in trypsin buffer (40 mM ammonium-bicarbonate in 9% acetonitrile/91% water)) overnight at 37ºC. The supernatant was transferred to a sample vial before the gel piece was extracted a second time with 15 μl of 0.1% trifluoroacetic acid in water. The combined extracts were centrifuged and 25 μl of the supernatant were transferred to a sample vial to allow LC-MS (Liquid chromatography-mass spectrometry) analysis using an Easy-nLC 1000 (Thermo Scientific) system coupled to a Fusion Lumos Tribrid mass spectrometer (Thermo Scientific).

Peptides were trapped on an AcclaimPepmap 100 C18 3 µm, 0.075 × 20 mm (Thermo Scientific) trap column and separated on a Thermo AcclaimPepmap RSLC C18 2 µm, 0.075 × 150 mm analytical column, using a gradient from 97% A (0.1% formic acid) to 35% B (0.1% formic acid in CAN) over 90 min, flow 0.3 µl/min. The mass spectrometer was operated in 3 s cycles where the MS spectra were recorded with the orbitrap analyzer at resolution 120,000 allowing the collection of up to 4e5 ions for maximal 50 ms before switching to MSMS mode. Multicharged ions (threshold 5e4) were fragmentated with equal priority by higher-energy collisional dissociation (HCD) (30% collision energy) and collision-induced dissociation (CID) (35% collision energy, 10 ms activation, Q 0.25) using quadrupole isolation with 1.6 Da width and 21 s dynamic exclusion. HCD ions (up to 5e4 ions) were collected for max 200 ms in the orbitrap analyzer at a resolution of 15,000. CID ions were recorded in the ion trap (rapid mode) aiming at higher sensitivity (threshold 1e4).

### Bioinformatics

To identify proteins tandem mass spectra data was analyzed with PEAKS software (version 10.6) against human and bacterial proteins in UniProt Swissprot and UniProt trEMBL databases (version v2022_03). Database search parameters were set as follows: precursor mass tolerance of 10 ppm, fragment mass tolerance of 0.02 Da and a maximum of two missed trypsin cleavages. False Discovery Rate (FDR) for both peptide and protein identifications was set to 1.0 %. Static modification was set to carbamidomethyl of cysteine, and variable modification to oxidation of methionine. A protein was considered identified if it was presented with at least one unique peptide and the total protein coverage of the supporting peptides was ≥ 1 %. Negative controls (PBS washes of unused disposable gloves) were analyzed in parallel with total sweat, EV-derived and patch-bound samples. All the proteins identified in negative control samples were excluded from the further protein analysis. These contaminants included some of the most abundant proteins of human sweat such as dermcidin and albumin 41 as well as multiple keratins (Supplementary Table 1). Protein identifications were processed in RStudio (version 2022.07.2, R version 4.2.2) and with package ggplot2 (version 3.4.0).

Functional classification of human proteins was performed using the PANTHER (Protein ANalysis THrough Evolutionary Relationships) Classification System (www.pantherdb.org) 42. Statistical analysis for PANTHER protein classes was performed in GraphPadPrism 10.0.2 using one-way ANOVA with post hoc Tukey's multiple comparisons test. Adjusted p < 0.05 was considered significant. Statistical analysis in PANTHER overrepresentation test was done with Fisher's exact test using the Bonferroni correction for multiple testing. GO (Gene Ontology) annotations with Bonferroni-corrected p < 0.05 and enrichment fold ≥ 10 were visualized in scatterplots with REViGO (http://revigo.irb.hr/) 43. For bacterial proteins, GO annotations were retrieved with Retrieve/ID mapping tool (https://www.uniprot.org/uploadlists/) from UniProt database and annotated manually to ancestor GO terms 44. Venn diagrams were prepared using Venny (version 2.1) (http://bioinfogp.cnb.csic.es/tools/venny/).

The mass spectrometry proteomics data have been deposited to the ProteomeXchange Consortium via the PRIDE 45 partner repository with the dataset identifier PXD045589.

## Results

### Characterization of EVs in human sweat

**Figure 1:**
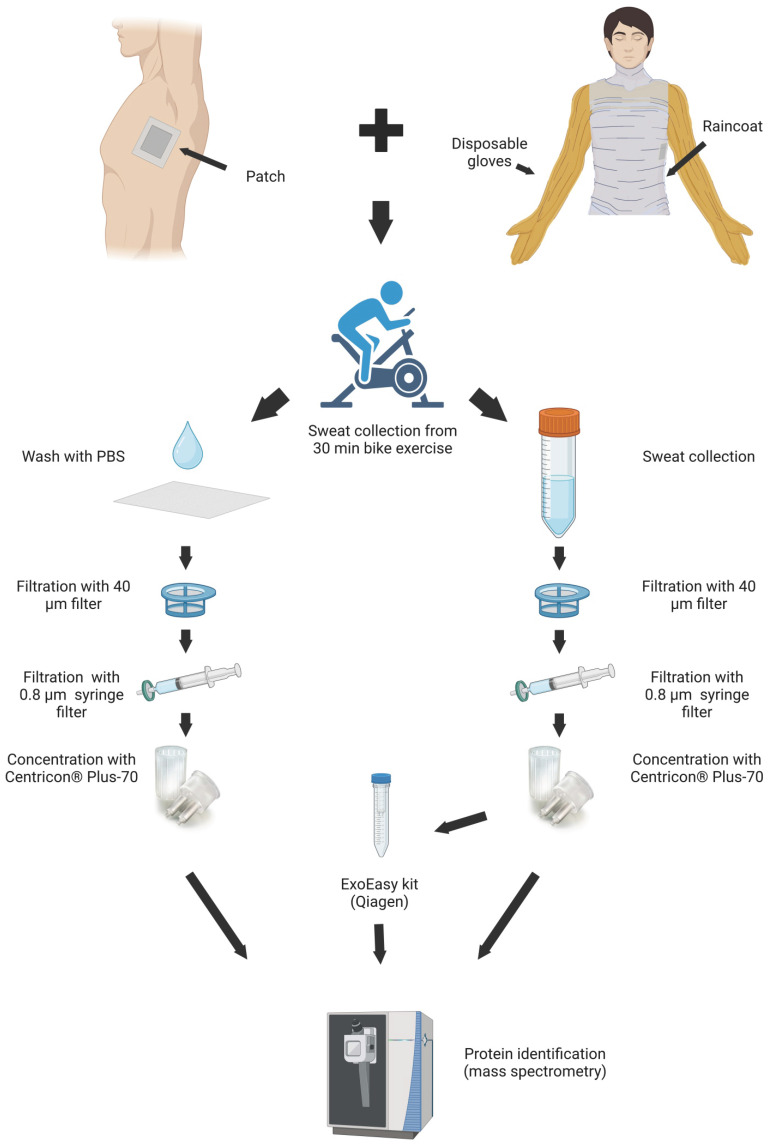
** Workflow of EV isolations from sweat.** Image created with BioRender.com.

Sweat samples were collected from eight volunteers undergoing vigorous biking exercise for 30 min, as described previously 27. Half of each sweat sample was used for EV isolation with an ExoEasy kit, and another half was kept to represent total sweat (Figure 1). During the same exercise volunteers carried skin patches, which were processed as described in Materials and Methods. The samples from each individual were analyzed separately.

**Figure 2:**
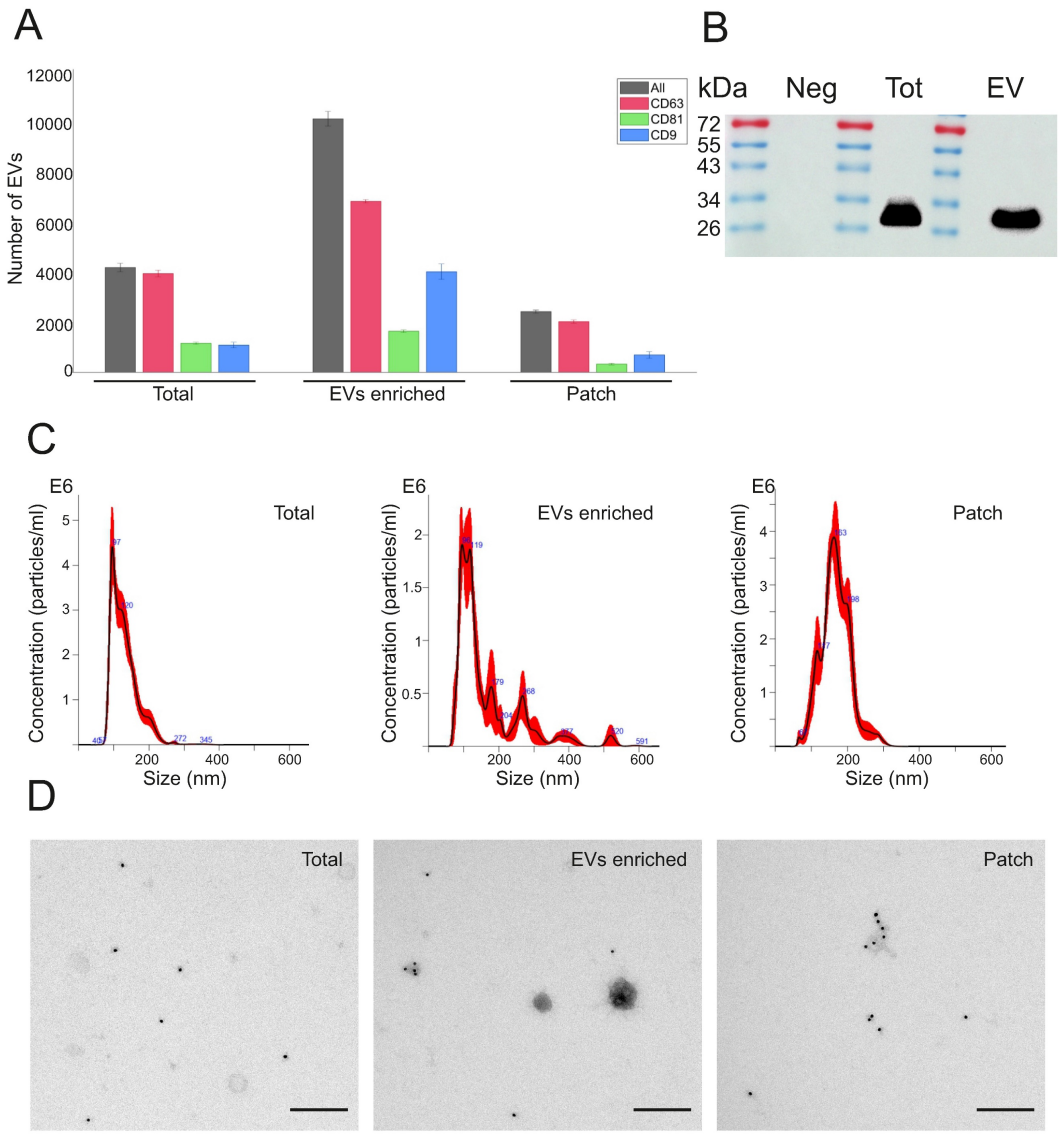
** Characterization of EVs present in total sweat with EV-enriched sweat samples and skin patches.** (A) Analysis of sweat EVs with ExoView platform. ExoVIew chip carrying CD63 antibody was used. Total numbers of detected EVs in each sample (1 µg total protein) are shown by black bars, number of EVs expressing CD63, CD81, and CD9 is shown by red, green, and blue bars, correspondingly. Data represent mean ± SEM from n = 3 slots on a chip. (B) Western blot analysis with anti-CD63 antibodies. Neg - Negative control (PBS after washing of glove and processed the same as sweat sample), Tot - total sweat, EV - EV enriched sample. Images of uncropped blots are shown in Supplementary Figure 4. (C) EV concentrations and size distribution measured by NTA. Dilutions used: 1:1000 for total sweat and EV-enriched samples, 1:100 for patch sample. (D) Immuno-TEM with anti-CD63 antibody (magnification 1:18,500).

The presence of EVs expressing typical exosomal markers CD63, CD81 and CD9 was analyzed in sweat by using ExoView R100 platform. When equal amount (1 µg) of protein was loaded into each chip, ExoView chips functionalized with anti-CD63 antibody (Figure 2A, Supplementary Figure 2A) bound higher number of EVs in all types of sweat samples (total, EVs enriched and patch) as compared to chips carrying anti-CD9 and anti-CD81 antibodies (Supplementary Figure 2B,C). These results correspond well to the previous findings that CD63 is the most abundant tetraspanin marker in sweat EVs 27,28. The total number of EVs captured on CD63 chips in skin patch samples comprised about 24% of EVs from EV enriched sweat and about 58% of concentrated sweat (Figure 2A). The mean sizes of sweat EVs, according to ExoView analysis, were 55 ± 8 nm. in total sweat, 68 ± 26 nm in EV enriched samples, and 57 ± 16 nm in sweat sample collected with patch (Supplementary Figure 3A). Comparison of EV samples isolated from different volunteers did not show much difference between samples, with CD63, CD81 and CD9 positive vesicles constituted, correspondingly, 58-66.5%, 11.6-26.4% and 39-49% of total number of nanoparticles in EV enriched samples (Supplementary Figure 3B). The presence of CD63 exosomal marker in total sweat and EV enriched preparations was confirmed by Western blotting (Figure 2B; Supplementary Figure 4).

Number and size distribution of EVs were measured by NTA. Average particle/ml concentration in total sweat samples was 2.68e+11 ± 1.58e+10 particles/ml. The amount of EVs in EV-enriched samples was 1.59e + 11 ± 1.20e+10 particles/ml, with lower numbers found in patch samples (3.38e+10 ± 4.10e+08 particles/ml) (Figure 2C). Total sweat was characterized by one distinct peak of EVs with the mean size of 130.8 ± 3.6 nm. In EV-enriched samples, EVs had mean size of 163 ± 12.3 nm, with a main peak at 96-119 nm and several smaller peaks at about 179 nm and 268 nm. Patch EVs were characterized by a similar mean size of 168.4 ± 2.9 nm, but more even distribution as compared to EV-enriched samples with the single peak at 163 nm (Figure 2C).

Concentrated total sweat and EV-enriched sweat under TEM displayed well-defined EVs in the size range of 30 to 200 nm (Figure 2D). Patch samples usually had lower number of EVs, but also less non-EV contaminant particles visible. Immunoelectron microscopy showed that many EVs expressed CD63 marker. In general, the obtained data confirmed that isolation method we used in the study results in purification of high amount of EVs from human sweat. Calcium alginate patches accumulate enough CD63-positive EVs to be detected by immunoelectron microscopy and ExoView EV analysis platform.

### Human proteins in sweat EVs

**Figure 3:**
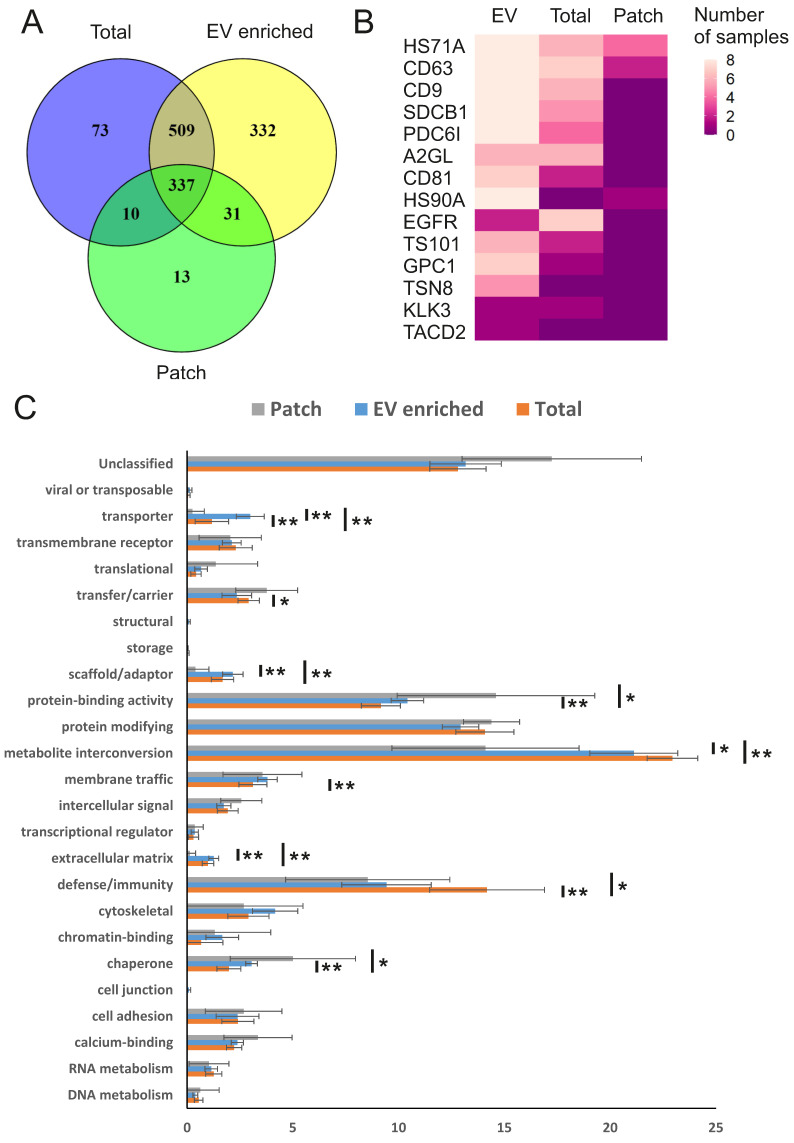
** Human proteins in sweat EVs.** (A) Venn diagram comparing human proteins identified in all individual sweat patch, EV-enriched and total (concentrated) sweat samples. All three types of samples were collected from 8 volunteers during the same biking exercise. (B) Number of samples in which selected proteins typical for EVs or associated with cancer were identified. HS71A: HSP70-1A; CD63: CD63 antigen; CD9: CD9 antigen; SDCB1: Syntenin-1; PDC6I: ALIX; A2GL: LRG; CD81: CD81 antigen; HS90A: HSP90-alpha; EGFR: Epidermal growth factor receptor; TS101: ESCRT-I complex subunit TSG101; GPC1: Glypican-1; TSN8: Tetraspanin-8; KLK3: PSA; TACD2: Tumor-associated calcium signal transducer 2. (C) Distribution of proteins by molecular classes. Proteins were analyzed using the PANTHER (http://www.pantherdb.org/). Each bar represents individual class, error bars show SEM for different samples. The percent share of the total for each molecular class is shown. All molecular classes identified in samples and statistical analysis results are shown in Supplementary Table 3. * p ≤ 0.01, ** p ≤ 0.05.

We next performed mass spectrometry analysis of individual sweat samples: total, EV-enriched, and patch-derived, to find out their protein composition. In total, 1305 unique human proteins were identified (Figure 3A; PEAK search raw data are shown in Supplementary Table 1; full list of identified human proteins shown in Supplementary Table 2). Of the total, 1209 proteins were present in EV-enriched samples, with approximately 70% of EV proteins in common with total sweat (846 proteins) and about 30% with patch samples (368 proteins). The numbers of proteins identified exclusively either in total sweat (83) or patch (23) were rather low. As expected, each of the analyzed EV-enriched sweat samples contained typical exosomal markers CD63, CD9, ALIX (ALG-2-interacting protein 1), syntenin-1, annexin A5, HSP90 (Heat shock protein 90 kDa) and HSP70 (Heat shock 70 kDa protein) 12,13, and the majority of these samples (6-7 out of 8) contained CD81 and TSG101 (Tumor susceptibility 101) (Figure 3B, Supplementary Table 2). In some samples we also identified several EV proteins that were previously suggested to be liquid biopsy markers for lung (LRG1 (Leucine-rich alpha-2-glycoprotein), tetraspanin-8), prostate (PSA (prostate-specific antigen)), bladder (TACD2 (Tumor-associated calcium signal transducer 2)), colorectal and pancreatic (glypican-1) cancers (reviewed by 46) (Figure 3B). The majority of the same proteins were also found in total concentrated sweat, though in lower number of samples. As for sweat collected with patches, it contained only CD63, the most abundant marker of sweat EVs, as well as heat shock proteins 70 and 90.

We used PANTHER (http://pantherdb.org/) 42 to group the sweat EV proteins into molecular classes (Figure 3C, Supplementary Table 3). The most represented classes were enzymes, which were either metabolite interconversion enzymes (21.1±2.1%), including hydrolases (PC00121), oxidoreductases (PC00176) and transferases (PC00220), or protein modifying enzymes (12.9±0.9%), which were predominantly proteases (PC00190). Also, protein-binding activity modulators (10.4±0.8%) and defense/immunity proteins (9.4±2.1%) such as immunoglobulins (PC00123) were found highly represented in the sweat EVs, while no other molecular class comprised more than 5% of the total protein number. There was little difference in protein classes distribution between total, EV-enriched and patch-collected sweat, indicating that sweat EVs could be used to reliably assess composition of the whole sweat. Most notably, defense/immunity proteins were significantly more abundant in total sweat samples (14.2±2.7%), while protein-binding activity modulators (14.6±4.7%) were overrepresented and metabolite interconversion enzymes (14.1±4.4%) underrepresented in patch samples.

In accordance with our data on protein molecular classes, Gene Ontology (GO) analysis for “biological processes” (https://geneontology.org/; summarized and visualized at http://revigo.irb.hr/) showed “metabolic process” (GO:0008152) and “cellular process” (GO:0009987) to be most common for total sweat, sweat EVs and patch samples (Supplementary Table 4). Overrepresentation test demonstrated that processes related to ESCRT (endosomal sorting complex required for transport) disassembly and viral budding were most enriched for total and EV-enriched sweat samples (Supplementary Table 4; Supplementary Figure 5). In addition, EV-enriched samples had strong overrepresentation for proteins involved in positive regulation of exosomal secretion (GO:1903543; 11.45-fold, p= 3.49E-03). Patch samples were enriched for proteins involved into several metabolic processes, protein refolding and antibacterial humoral response.

GO analysis for “cellular components” showed that the most common “Cellular anatomical entities” for all sample types (total, EV-enriched and patch) were membrane proteins and intracellular structures (Supplementary Table 4). Overrepresentation tests found proteasomal complexes to be most strongly enriched in total sweat and sweat EVs, while patch samples also were highly enriched for IgA immunoglobulin complexes (Supplementary Table 4; Supplementary Figure 6).

GO analysis for “molecular function” showed that the majority of proteins from all types of samples (total, EV-enriched and patch) were involved either in catalytic activity or binding (predominantly protein binding) (Supplementary Table 4). In overrepresentation tests we observed highest enrichment of sweat EV proteins for mannosidase activity, total sweat proteins for chloride ion binding and interleukin-1 receptor binding, and patch proteins for several enzymatic pathways and myosin V binding (Supplementary Table 4; Supplementary Figure 7).

**Figure 4:**
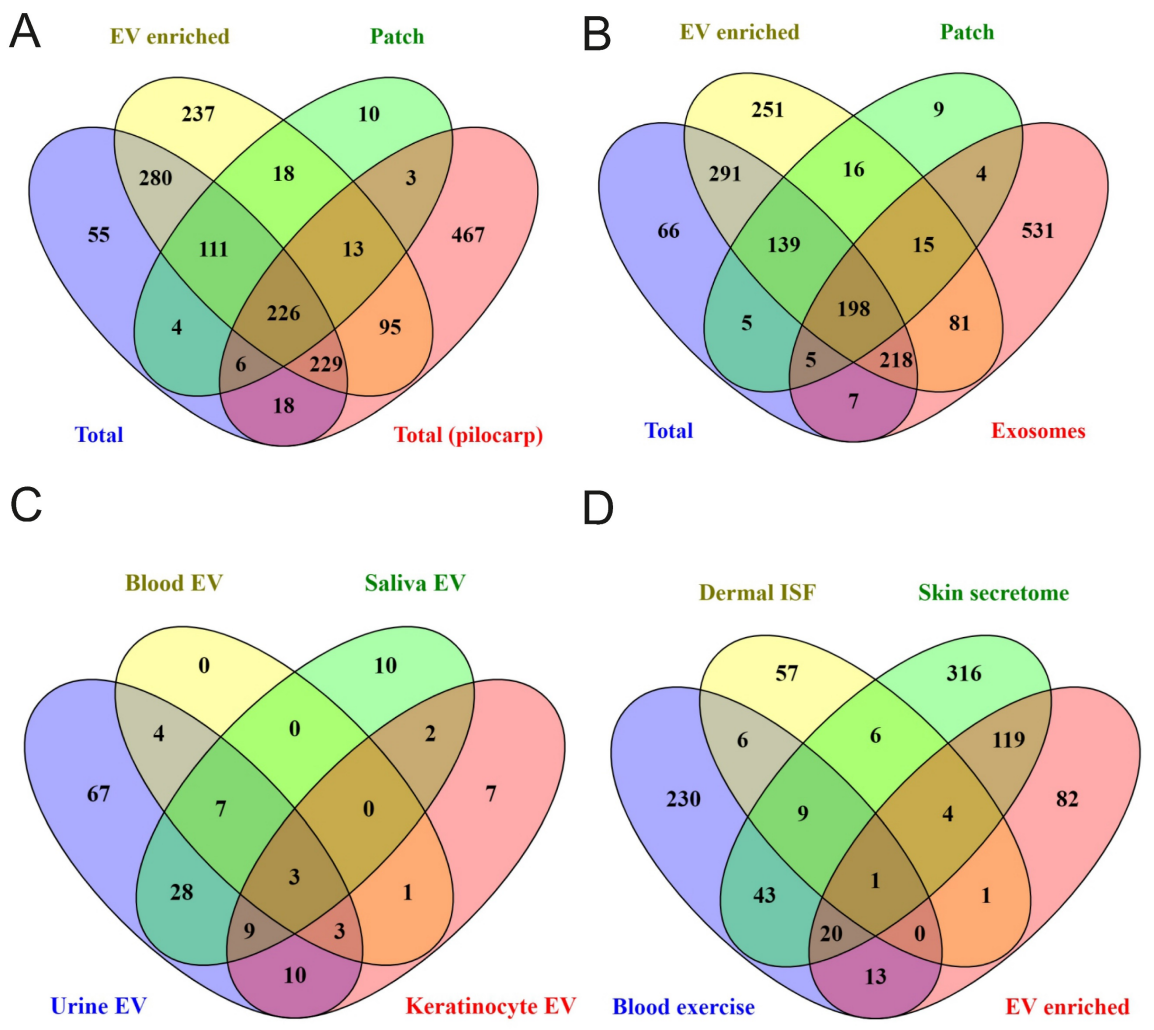
** Comparison of obtained data with previous proteomics studies results.** Comparison of sweat proteins identified in our study with (A) proteins described in total sweat upon pilocarpine stimulation (“Total (pilocarp)”) 35 and (B) in sweat exosomes (”exosome”) 37. (C) Comparison of proteins found in all EV-enriched sweat samples with ExoCarta data for human biofluids' and keratinocytes' EVs (http://www.exocarta.org). (D) Venn diagram comparing “core” sweat EV proteins identified in our study with skin washes (”skin secretome”), dermal interstitial fluid (”Dermal ISF”) (only proteins significantly higher expressed in ISF compared to plasma are analyzed), and blood EVs (only proteins significantly induced in plasma by exercise are analyzed) (”Blood exercise”) 47-49. Actual protein lists used for comparisons are given in Supplementary Table 5.

To find out how well sweat protein composition identified in the present study reflects protein composition of sweat in general, we made comparisons with published data. The number of sweat proteins identified in different studies vary widely (comparison of sweat proteins from various publications are shown in 34). Comparison of our data with a recent study of sweat secretion stimulated by pilocarpine iontophoresis, which described one of the highest number of sweat proteins 35, demonstrated that out of 1057 proteins in pilocarpine-induced sweat 479 were shared with “total sweat proteins” from our study (Figure 4A; Supplementary Table 5). In addition, 108 proteins from 35 were identified only in EV-enriched sweat but not in total sweat samples.

We next compared the proteomics data from our study to a previously published study, in which exosomes were isolated by sucrose density gradient centrifugation from pooled sweat of healthy adult volunteers performing aerobic exercise 37 (Figure 4B; Supplementary Table 5). 697 EV proteins from our study were not described in that sweat exosomal study and 531 proteins from sweat exosomes were not found in any type of sweat samples (EV-enriched, total and patch) we analyzed. Only 512 proteins were common for EV enriched sweat we describe and sweat exosomes from the previous study 37, from which 198 proteins were shared between EV-enriched, total, patch sweat and sweat exosome proteome (Figure 4B). The difference can be explained by different sweat collection and EV isolation protocols, but also may reflect a high variability of proteins between individual samples.

Comparison of samples from different individuals allows to define “core” sweat EV proteins, that were identified in each sample. It turned out that the “core” includes 240 proteins (about 20% of all identified EV proteins), among them many well-known exosomal markers (Figure 3B, Supplementary Tables 2 and 6). Out of 240 most common sweat EV proteins, 154 were present in sweat exosomes as described by 37 and 183 were already described in EVs from at least one human biofluid (as reported in ExoCarta database; http://www.exocarta.org/)). The majority of these proteins were shown in exosomes isolated from urine (131 out of 240), but also blood (plasma and/or serum), saliva, breast milk and other biofluids (Figure 4C, Supplementary Table 6). Sweat EVs had much more common proteins with urine exosomes (ca. 55%) than with EVs secreted by cultured keratinocytes (ca. 15%) (Figure 4C, Supplementary Table 6). 165 out of 240 “core” proteins were bound by skin patches (Supplementary Table 2). Like EV-enriched sweat in general, patch EV proteins also showed a lot of similarities with biofluids' EVs, as well as some proteins typical for keratinocytes' EVs (Supplementary Figure 8A).

We also included in comparison the results of 49, which instead of collecting sweat produced during physical exercise, analyzed composition of wash solution incubated with skin for 2 minutes (so called “skin secretome”). 144 proteins (ca. 60%) were common between our “core” sweat EV data and skin secretome (Figure 4D). One possible source of EVs in sweat may be dermal ISF, but comparison of our sweat EV data with the list of 84 proteins specific for dermal ISF found only 6 common ones 48 (Figure 4D). 34 sweat EV proteins, including several annexins (A1, A3, A5, A11) and calpain-1 catalytic subunit, were significantly upregulated in serum EVs in response to exercise 47 (Figure 4D). Similarly to EV-enriched sweat, patch samples contained multiple proteins, characteristic of skin secretome, as well as 46 proteins shown to be upregulated in blood EVs by exercise 47,49 (Supplementary Figure 8B).

In general, we can conclude that the protein composition of sweat EVs, either isolated by ExoEasy columns or bound to alginate patches, is mostly similar with the composition of total sweat, and it contains multiple proteins that are typical for other human biofluids.

### EV glycosylation patterns

Since changes in glycosylation patterns are commonly seen in cancer cells, glycoproteins are often used as prognosis cancer biomarkers 50. Until recently, little attention has been given to the protein glycosylation patterns of EVs, though it is important for vesicles biogenesis, uptake and cargo recruitment 51,52. In order to characterize glycosylation of sweat EVs we employed lectin microarray analysis. We found that sweat derived EVs contain N- and O-glycosylated proteins carrying mainly galactose-ending N-glycans and fucosylated N-glycans (Supplementary Figure 9, Supplementary Table 7). The O-glycosylated glycans were truncated and displayed only the initiating N-acetyl galactosamine (GalNAc) and the mucin type core 1 structure (Gal-β(1,3)-GalNAc). Identified in other types of EVs WGA (wheat germ agglutinin) -binding glycans ((GlcNAcβ4)n) as well as MALI and MALII (*Maackia amurensis* lectins I and II) binding glycans (α-2,3-sialylated N-glycans) were not detected 51,52.

We also found differences in glycosylation between individual samples, especially visible for binding of Galα(1,3)Gal or Galα(1,3)Galβ(1,4)GlcNAc to *Marasmium oreades* agglutinin (MOA). This variation probably stems from the ability of this lectin to bind specifically terminal Galα(1,3)Gal residues present typically in the xenotransplantation epitope (Galα(1,3)Galβ(1,4)GlcNAc) and the branched blood group B determinant (Galα(1,3)[Fucα(1,2)]Gal) 53.

### Microbiome-derived proteins in sweat EVs

**Figure 5:**
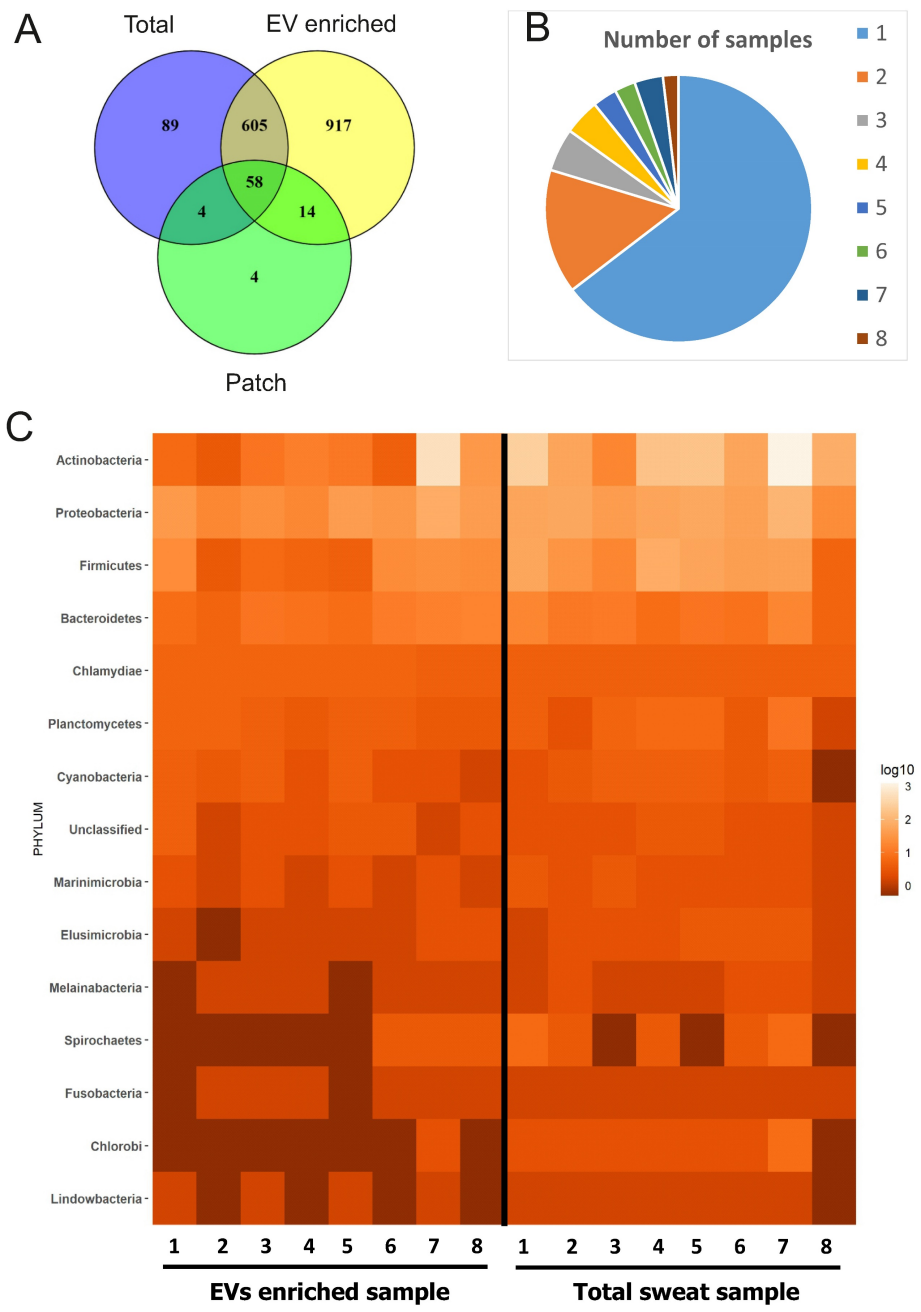
** Bacterial proteins in sweat EVs.** (A) Venn diagram comparing proteins of bacterial origin identified in sweat patches, EV-enriched samples and total (concentrated) sweat. (B) Frequency of occurrence of bacterial proteins in individual samples. Each color shows how many different proteins were found either in single samples (1), multiple samples (2-7), or all samples studied (8). The full list of proteins is given in Supplementary Table 8. (C) Distribution of proteins identified in individual sweat EV samples between bacterial phylae (see also Supplementary Table 9). Each column represents an individual sample. Color indicates how many different proteins from the same order are found in the sample. Number of protein hits assigned to each phylum in each sample are presented as log10-score.

Since human skin is permanently populated by diverse microorganisms 17 and bacterial EVs such as outer membrane vesicles (OMV) are increasingly recognized and characterized 14,54,55, we investigated the contribution of the skin microbiome to sweat and EV-enriched sweat protein composition. 1691 proteins of bacterial origin were identified from all samples, which is more than the number of human proteins (Supplementary Table 8; Figure 5A). EV-enriched sweat samples contained all together 1594 bacterial proteins, and total sweat samples 756. Individual variability of bacterial proteins between samples was higher than for human proteins with the majority of proteins only detected in single samples (1030 out of 1594), and only about 3% (30 proteins) could be detected in all 8 samples studied (Figure 5B). Interestingly, unlike with human proteins, there were few proteins of bacterial origin (80) found on skin patches (Figure 5A).

In respect to taxonomy, the composition of EV-enriched sweat bacterial proteins was mostly similar to the composition of total sweat samples (Figure 5C, Supplementary Figure 10A). The majority of bacterial proteins in EV-enriched sweat belonged to phyla *Actinobacteria,* followed by *Proteobacteria* and *Firmicutes* (Figure 5C). Interestingly, proteins derived from phyla *Bacteroidetes*, typical colonizers of the gut, were also identified in sweat EVs. Taxonomical distribution of bacterial proteins in sweat EVs varied highly between individuals, probably reflecting individual differences in bacterial communities populating skin (Figure 5C). For example, proportion of proteins from *Actinobacteria* varied between 15% and 85% of all identified bacterial proteins depending on the sample. The largest proportion of patch-bound bacterial proteins belonged to *Proteobacteria*, which probably reflects specific bacterial composition of skin site where patches were located (Supplementary Table 9) 56,57. Many genera identified by proteomics (*Cutibacterium, Corynebacterium, Staphylococcus, Micrococcus, etc.*) are typical components of the skin microbiota 17. Some genera include well known pathogenic bacteria causing skin infections (*Mycobacterium, Cutibacterium, Staphylococcus, Campylobacter, etc.*).

**Figure 6:**
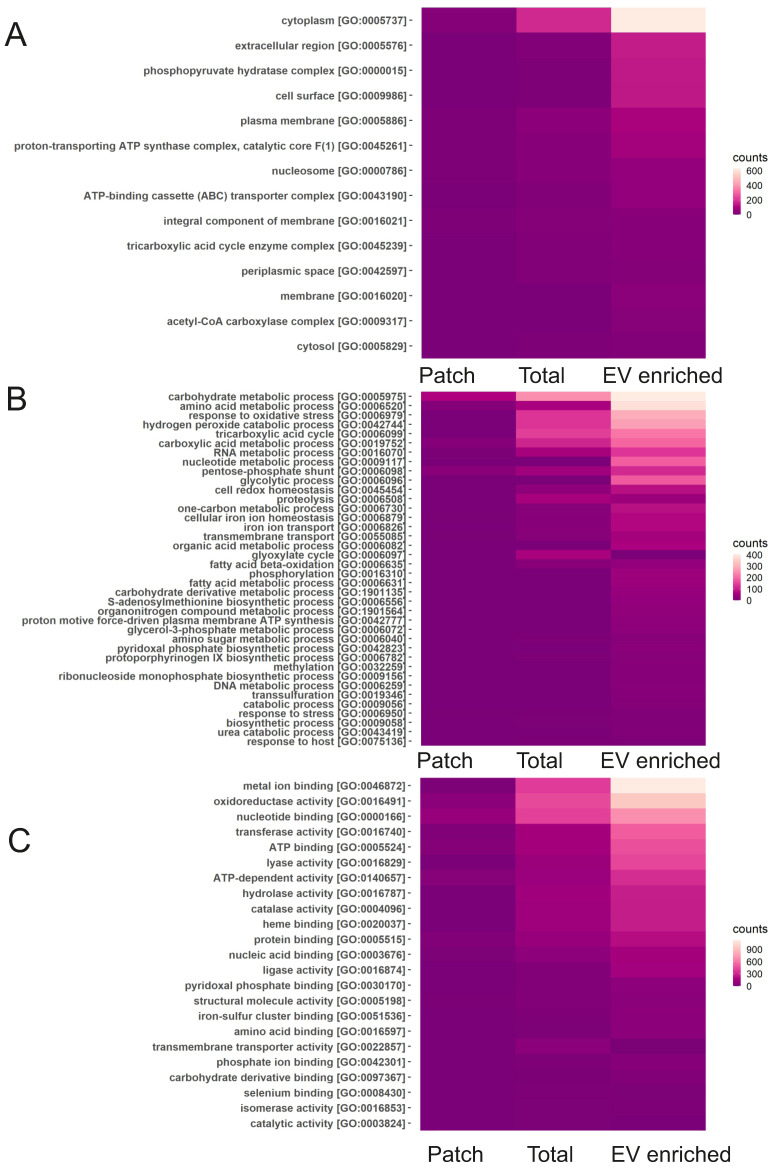
** Classification of bacterial sweat EV proteins by GO annotations.** Analysis of bacterial proteins found in total, EV enriched and patch sweat samples for biological processes for cellular components (A), biological processes (B) and molecular function (C).

Among the most commonly found bacterial proteins in sweat EVs were ubiquitin and Glyceraldehyde-3-phosphate dehydrogenase (EC 1.2.1.-), which contribute to virulence of many pathogenic bacteria 58. GO analysis for “cellular components” showed that the majority of identified bacterial proteins are cytoplasmic or represent extracellular region (Figure 6A). Based on GO analysis, highly enriched “molecular functions” for bacterial proteins are metal ion binding, nucleotide binding as well as oxidoreductase activity, and “biological process” represented different metabolic processes and oxidative stress response (Figure 6B, C). GO annotations of EV-enriched sweat and total sweat bacterial proteins were mostly similar.

## Discussion

Unlike other biofluids, sweat EVs have not been extensively investigated 27,28,37. The potential limitations due to low concentration of EVs in sweat and high variability of sweat content between individuals may be the main reason for that. Our own data on total sweat composition showed rather good overlap with published studies 3,4,11,34-36, but also found previously unreported proteins (Figure 4). Sweat composition is highly dynamic depending on the metabolism with temperature and muscular activity affecting amount of liquid, miRNAs, ionic and metabolite concentrations. Therefore, sweat analysis results depend on the methods used for collecting sweat. All together three different strategies to stimulate sweat production for proteomics studies were tested in humans 2,34: pharmacological cholinergic stimulation by pilocarpine iontophoresis, temperature increase and physical exercise. Pilocarpine stimulation in combination with Macroduct collection system reflects “baseline” sweating, but it acts only via activation of one type of receptors (muscarinic receptors), applies to small areas of skin, results in low volumes of collected sweat and has relatively low accuracy due to the mixing of sweat and gel fluid 59. The method of sweat collection used in our study (biking exercise wearing gloves and raincoat) results in large volumes of sweat that contains mixture of sebum, eccrine and apocrine sweat from various parts of body. It includes components induced by muscular activity as well as by external heating of the body.

The only publication describing proteomics of sweat-derived exosomes 37 used pooled sweat sample from 13 participants exercising in hot weather (35 °C). Isolation of small EVs using a gradient ultracentrifugation from individual samples turned out to be not feasible, however, because the yields were too low (Zhyvolozhnyi and Samoylenko, unpublished observation). Instead of small EVs, we focused on a broader range of sweat EVs, isolated by size-exchange chromatography using ExoEasy columns (Figure 1). We identified the protein composition of individual EV-enriched sweat samples under conditions similar to the ones we used to analyze RNA content of EV-enriched sweat 27,28.

After analyzing the protein content of all EV samples, we defined a “sweat EV core” consisting of 240 proteins, that were identified in all samples tested. Typical exosomal markers such as CD63, CD9, ALIX, syntenin-1, annexin A5, HSP90, HSP70 were all identified among these sweat EV “core” proteins. Also, we found some potential cancer markers, such as LRG1, tetraspanin-8, PSA, TACD2 and glypican-1 46 (Figure 3B). Among these markers PSA may be of special interest since serum PSA detection is the most common method for initial prostate cancer screening. Other clinically proven disease protein biomarkers identified in EV-enriched sweat were C-reactive protein, a widely used inflammation marker detected by blood tests, alanine aminotransferase (ALT), aspartate transaminase (AST) and lactate dehydrogenase (LDH) 60. ALT is an enzyme that is primarily found in liver cells, and its release into the bloodstream is often associated with liver damage 61. AST, like ALT, is mostly found in liver but is also present in cardiac and skeletal muscle cells. Increased AST levels in the bloodstream are indicative of tissue damage or injury. LDH is an enzyme involved in cellular metabolism and is found in various tissues, including the heart, liver, and skeletal muscles. Elevated levels of LDH in circulation are often associated with tissue damage or cell death 62. The presence of ALT, AST and LDH in EV-enriched sweat suggests that sweat EVs have potential for assessing liver and cardiac health as well as musculoskeletal disorders through non-invasive means.

We observed a lot of similarities between protein composition of sweat EVs and previously reported data describing EVs from other human biological fluids, especially urine (Figure 4B). It is not possible to conclude whether all the identified sweat EV proteins are directly derived from skin and sweat glands or whether some EVs from internal organs are transported in blood and transferred to sweat by the filtration system. A UniProt database search illustrated that the majority of identified sweat EV proteome components are ubiquitously expressed. Some are usually found in skin and/or mucosa such as serpin B13, kallikrein-7, and skin-specific protein 32, reinforcing the value of the EVs in skin biology. Several identified proteins were present in EVs from keratinocyte culture, but not reported in biofluids before: NADP-ME1 (NADP-dependent malic enzyme), DPP3 (Dipeptidyl peptidase 3), GC (Group-specific component), DPP2 (Dipeptidyl peptidase 2), VCAN (Versican core protein), ALDH7A1 (Aldehyde dehydrogenase family 7 member A1), LFNG (Beta-1,3-N-acetylglucosaminyltransferase lunatic fringe). Typical blood proteins such as the complement system components and hemoglobin subunits, neuronal protein (neuroserpin), testis proteins (semenogelins 1 and 2), and bone marrow protein Neutrophil elastase (EC 3.4.21.37) 63 were all found in EV-enriched sweat. These observations suggest that sweat EVs may carry material derived not only from skin but also from other organs. In a similar way EVs from prostate cancer cells could be found in urine 64,65.

Studies of bacteria-derived EVs such as OMV, in multiple processes including pathogenesis promotion or nutrients uptake, emerged in the last couple of years 54. It has been shown that OMVs can induce specific immune responses in the host 66. Our study is the first to report protein content of bacterial EVs in human sweat. Characterization of sweat microbiota EVs may be important for the analysis of skin bacterial infections as well as non-infectious diseases associated with the changes of skin bacteria composition, including diabetes mellitus 16. We have identified *Actinobacteria* as the most abundant phylum in sweat EVs, followed by *Proteobacteria* and *Firmicutes*. The comparison of these proteomics results with our previously reported analysis of sweat EVs based on nucleic acids 27 showed higher proportion of *Actinobacteria* and lower proportion of *Proteobacteria*. In general, taxonomic diversity of bacterial sweat EVs proteins was well correlated with the data of total sweat and reflected high variability of skin microbiota composition between individuals.

Large amounts of sweat (up to several hundred milliliters) required for isolating vesicles in our study by using ExoEasy columns is an important factor limiting its possible clinical use. Therefore, development of small size user-friendly and cost-effective sweat collectors is highly needed. Alginate-based hydrogels were previously used to encapsulate and deliver EVs for treatment of myocardial infarction 67 and for diabetic wound healing 23. Our report is the first to show that alginate skin patches could be used for studying protein composition of EVs. Though many typical EV markers were not detected in the patch samples, they contained CD63, which was shown before to be the main EV marker in sweat 27,28. Interestingly, alginate patches collected very little proteins of bacterial origin. This may indicate differences in binding of EVs with distinct membrane composition to the skin patches used in the study. Our results may provide a solution for separation of human EVs from bacterial EVs, that presents a serious challenge in human biofluids and bacteria-rich samples such as feces 68.

In conclusion, we identified proteins that are present across different individual samples of human sweat and showed potential biomarkers for cancer and infectious diseases among sweat EV proteins. Our data demonstrated that skin patches could be used for collection of sweat EVs of human origin and their separation from bacterial EVs.

## Supplementary Material

Supplementary figures and tables 3 and 7, table legends.

Supplementary tables 1, 2, 4, 5, 6, 8, 9.

## Abbreviations

ALIX: ALG-2-interacting protein 1
ALT: alanine aminotransferase
AST: aspartate transaminase
CID: collision-induced dissociation
EGFR: Epidermal growth factor receptor
ESCRT: endosomal sorting complex required for transport
EVs: extracellular vesicles
GO: Gene Ontology
HCD: higher-energy collisional dissociation
HSP90: Heat shock protein 90 kDa
HSP70: Heat shock 70 kDa protein
ISF: interstitial fluid
LC-MS: Liquid chromatography-mass spectrometry
LDH: lactate dehydrogenase
LRG: Leucine-rich alpha-2-glycoprotein
NTA: Nanoparticle tracking analysis
OMV: outer membrane vesicles
PAA: polyacrylamide
PANTHER: Protein ANalysis THrough Evolutionary Relationships
PSA: Prostate-specific antigen
TACD2: Tumor-associated calcium signal transducer 2
TEM: transmission electron microscopy
TSG101: Tumor susceptibility 101

## References

[1] Lin J, Ma L, Zhang D (2019). Tumour biomarkers—Tracing the molecular function and clinical implication. Cell Proliferation.

[2] Yuan X, Li C, Yin X (2023). Epidermal Wearable Biosensors for Monitoring Biomarkers of Chronic Disease in Sweat. Biosensors (Basel).

[3] Raiszadeh MM, Ross MM, Russo PS (2012). Proteomic analysis of eccrine sweat: Implications for the discovery of schizophrenia biomarker proteins. J Proteome Res.

[4] Adewole OO, Erhabor GE, Adewole TO (2016). Proteomic profiling of eccrine sweat reveals its potential as a diagnostic biofluid for active tuberculosis. Proteomics Clin Appl.

[5] Cui X, Zhang L, Su G, Kijlstra A, Yang P (2021). Specific sweat metabolite profile in ocular Behcet's disease. Int Immunopharmacol.

[6] Zhang X, Xia Y, Liu Y, Mugo SM, Zhang Q (2022). Integrated Wearable Sensors for Sensing Physiological Pressure Signals and β-Hydroxybutyrate in Physiological Fluids. Anal Chem.

[7] Karpova E V, Shcherbacheva E V, Galushin AA, Vokhmyanina D V, Karyakina EE, Karyakin AA (2019). Noninvasive diabetes monitoring through continuous analysis of sweat using flow-through glucose biosensor. Anal Chem.

[8] Cuartero M, Parrilla M, Crespo GA (2019). Wearable potentiometric sensors for medical applications. Sensors (Switzerland).

[9] Katchman BA, Zhu M, Blain Christen J, Anderson KS (2018). Eccrine Sweat as a Biofluid for Profiling Immune Biomarkers. Proteomics Clin Appl.

[10] Mena-Bravo A, Luque de Castro MD (2014). Sweat: A sample with limited present applications and promising future in metabolomics. Journal of Pharmaceutical and Biomedical Analysis.

[11] Harshman SW, Pitsch RL, Smith ZK (2018). The proteomic and metabolomic characterization of exercise-induced sweat for human performance monitoring: A pilot investigation. PLoS One.

[12] Van Niel G, D'Angelo G, Raposo G (2018). Shedding light on the cell biology of extracellular vesicles. Nat Rev Mol Cell Biol.

[13] Dixson AC, Dawson TR, Di Vizio D, Weaver AM (2023). Context-specific regulation of extracellular vesicle biogenesis and cargo selection. Nature Reviews Molecular Cell Biology.

[14] Avila-Calderón ED, Araiza-Villanueva MG, Cancino-Diaz JC (2015). Roles of bacterial membrane vesicles. Archives of Microbiology.

[15] Park KS, Lee J, Lee C (2018). Sepsis-like systemic inflammation induced by nano-sized extracellular vesicles from feces. Front Microbiol.

[16] Zhang S, Cai Y, Meng C (2021). The role of the microbiome in diabetes mellitus. Diabetes Research and Clinical Practice.

[17] Byrd AL, Belkaid Y, Segre JA (2018). The human skin microbiome. Nature Reviews Microbiology.

[18] Sempionatto JR, Lasalde-Ramírez JA, Mahato K, Wang J, Gao W (2022). Wearable chemical sensors for biomarker discovery in the omics era. Nature Reviews Chemistry.

[19] Bhide A, Muthukumar S, Prasad S (2018). CLASP (Continuous lifestyle awareness through sweat platform): A novel sensor for simultaneous detection of alcohol and glucose from passive perspired sweat. Biosens Bioelectron.

[20] Garcia-Rey S, Gil-Hernandez E, Basabe-Desmonts L, Benito-Lopez F (2023). Colorimetric Determination of Glucose in Sweat Using an Alginate-Based Biosystem. Polymers (Basel).

[21] Upasham S, Tanak A, Jagannath B, Prasad S (2018). Development of ultra-low volume, multi-bio fluid, cortisol sensing platform. Sci Rep.

[22] Currano LJ, Sage FC, Hagedon M, Hamilton L, Patrone J, Gerasopoulos K (2018). Wearable Sensor System for Detection of Lactate in Sweat. Sci Rep.

[23] Zhang Y, Zhang P, Gao X, Chang L, Chen Z, Mei X (2021). Preparation of exosomes encapsulated nanohydrogel for accelerating wound healing of diabetic rats by promoting angiogenesis. Materials Science and Engineering C.

[24] Shao H, Chung J, Lee K (2015). Chip-based analysis of exosomal mRNA mediating drug resistance in glioblastoma. Nat Commun.

[25] Liu F, Vermesh O, Mani V (2017). The Exosome Total Isolation Chip. ACS Nano.

[26] Lewis JM, Vyas AD, Qiu Y, Messer KS, White R, Heller MJ (2018). Integrated Analysis of Exosomal Protein Biomarkers on Alternating Current Electrokinetic Chips Enables Rapid Detection of Pancreatic Cancer in Patient Blood. ACS Nano.

[27] Bart G, Fischer D, Samoylenko A (2021). Characterization of nucleic acids from extracellular vesicle-enriched human sweat. BMC Genomics.

[28] Karvinen S, Sievänen T, Karppinen JE (2020). MicroRNAs in Extracellular Vesicles in Sweat Change in Response to Endurance Exercise. Front Physiol.

[29] Woodley FW, Gecili E, Szczesniak RD (2022). Sweat metabolomics before and after intravenous antibiotics for pulmonary exacerbation in people with cystic fibrosis. Respir Med.

[30] Brunmair J, Gotsmy M, Niederstaetter L (2021). Finger sweat analysis enables short interval metabolic biomonitoring in humans. Nat Commun.

[31] Cui X, Su G, Zhang L (2020). Integrated omics analysis of sweat reveals an aberrant amino acid metabolism pathway in Vogt-Koyanagi-Harada disease. Clin Exp Immunol.

[32] Schranner D, Kastenmüller G, Schönfelder M, Römisch-Margl W, Wackerhage H (2020). Metabolite Concentration Changes in Humans After a Bout of Exercise: a Systematic Review of Exercise Metabolomics Studies. Sports Medicine - Open.

[33] Rahat ST, Mäkelä M, Nasserinejad M (2023). Clinical-Grade Patches as a Medium for Enrichment of Sweat-Extracellular Vesicles and Facilitating Their Metabolic Analysis. Int J Mol Sci.

[34] Burat B, Reynaerts A, Baiwir D (2021). Characterization of the human eccrine sweat proteome—a focus on the biological variability of individual sweat protein profiles. Int J Mol Sci.

[35] Burat B, Reynaerts A, Baiwir D (2022). Sweat Proteomics in Cystic Fibrosis: Discovering Companion Biomarkers for Precision Medicine and Therapeutic Development. Cells.

[36] Yu Y, Prassas I, Muytjens CMJ, Diamandis EP (2017). Proteomic and peptidomic analysis of human sweat with emphasis on proteolysis. J Proteomics.

[37] Wu CX, Liu ZF (2018). Proteomic Profiling of Sweat Exosome Suggests its Involvement in Skin Immunity. Journal of Investigative Dermatology.

[38] Ferraraccio LS, Bertoncello P (2023). Electrochemiluminescence (ECL) biosensor based on tris(2,2′-bipyridyl)ruthenium(II) with glucose and lactate dehydrogenases encapsulated within alginate hydrogels. Bioelectrochemistry.

[39] Gunatilake UB, Garcia-Rey S, Ojeda E, Basabe-Desmonts L, Benito-Lopez F (2021). TiO _2_ Nanotubes Alginate Hydrogel Scaffold for Rapid Sensing of Sweat Biomarkers: Lactate and Glucose. ACS Appl Mater Interfaces.

[40] Ibarlucea B, Pérez Roig A, Belyaev D, Baraban L, Cuniberti G (2020). Electrochemical detection of ascorbic acid in artificial sweat using a flexible alginate/CuO-modified electrode. Microchimica Acta.

[41] Csosz Emri G, Kallõ G Tsaprailis G, Tozsér J (2015). Highly abundant defense proteins in human sweat as revealed by targeted proteomics and label-free quantification mass spectrometry. Journal of the European Academy of Dermatology and Venereology.

[42] Thomas PD, Ebert D, Muruganujan A, Mushayahama T, Albou LP, Mi H (2022). PANTHER: Making genome-scale phylogenetics accessible to all. Vol. 31, Protein Science.

[43] Supek F, Bošnjak M, Škunca N, Šmuc T (2011). Revigo summarizes and visualizes long lists of gene ontology terms. PLoS One.

[44] Ashburner M, Ball CA, Blake JA (2000). Gene ontology: Tool for the unification of biology. Nature Genetics.

[45] Perez-Riverol Y, Bai J, Bandla C (2022). The PRIDE database resources in 2022: A hub for mass spectrometry-based proteomics evidences. Nucleic Acids Res.

[46] Huang D, Rao D, Xi X, Zhang Z, Zhong T (2022). Application of extracellular vesicles proteins in cancer diagnosis. Front Cell Dev Biol.

[47] Whitham M, Parker BL, Friedrichsen M (2018). Extracellular Vesicles Provide a Means for Tissue Crosstalk during Exercise. Cell Metab.

[48] Tran BQ, Miller PR, Taylor RM (2018). Proteomic Characterization of Dermal Interstitial Fluid Extracted Using a Novel Microneedle-Assisted Technique. J Proteome Res.

[49] Burian M, Velic A, Matic K (2015). Quantitative proteomics of the human skin secretome reveal a reduction in immune defense mediators in ectodermal dysplasia patients. Journal of Investigative Dermatology.

[50] Martins ÁM, Ramos CC, Freitas D, Reis CA (2021). Glycosylation of cancer extracellular vesicles: Capture strategies, functional roles and potential clinical applications. Cells.

[51] Batista BS, Eng WS, Pilobello KT, Hendricks-Muñoz KD, Mahal LK (2011). Identification of a conserved glycan signature for microvesicles. J Proteome Res.

[52] Williams C, Pazos R, Royo F (2019). Assessing the role of surface glycans of extracellular vesicles on cellular uptake. Sci Rep.

[53] Grahn EM, Winter HC, Tateno H, Goldstein IJ, Krengel U (2009). Structural Characterization of a Lectin from the Mushroom Marasmius oreades in Complex with the Blood Group B Trisaccharide and Calcium. J Mol Biol.

[54] Gilmore WJ, Johnston EL, Zavan L, Bitto NJ, Kaparakis-Liaskos M (2021). Immunomodulatory roles and novel applications of bacterial membrane vesicles. Mol Immunol.

[55] Liu H, Geng Z, Su J (2022). Engineered mammalian and bacterial extracellular vesicles as promising nanocarriers for targeted therapy. Extracell Vesicles Circ Nucl Acids.

[56] Swaney MH, Nelsen A, Sandstrom S, Kalan LR (2023). Sweat and Sebum Preferences of the Human Skin Microbiota. Microbiol Spectr.

[57] Grice EA, Segre JA (2011). The skin microbiome. Nature Reviews Microbiology.

[58] Kopeckova M, Pavkova I, Stulik J (2020). Diverse Localization and Protein Binding Abilities of Glyceraldehyde-3-Phosphate Dehydrogenase in Pathogenic Bacteria: The Key to its Multifunctionality?. Frontiers in Cellular and Infection Microbiology.

[59] Wang M, Yang Y, Min J (2022). A wearable electrochemical biosensor for the monitoring of metabolites and nutrients. Nat Biomed Eng.

[60] Wishart DS, Bartok B, Oler E (2021). MarkerDB: An online database of molecular biomarkers. Nucleic Acids Res.

[61] Senior JR (2012). Alanine aminotransferase: A clinical and regulatory tool for detecting liver injury-past, present, and future. Clin Pharmacol Ther.

[62] Wu Y, Lu C, Pan N (2021). Serum lactate dehydrogenase activities as systems biomarkers for 48 types of human diseases. Sci Rep.

[63] Rumble JM, Huber AK, Krishnamoorthy G (2015). Neutrophil-related factors as biomarkers in EAE and MS. Journal of Experimental Medicine.

[64] Bernardino RMM, Leão R, Henrique R (2021). Extracellular vesicle proteome in prostate cancer: A comparative analysis of mass spectrometry studies. International Journal of Molecular Sciences.

[65] Dhondt B, Geeurickx E, Tulkens J (2020). Unravelling the proteomic landscape of extracellular vesicles in prostate cancer by density-based fractionation of urine. J Extracell Vesicles.

[66] Sivanantham A, Alktaish W, Murugeasan S, Gong B, Lee H, Jin Y (2023). Caveolin-1 regulates OMV-induced macrophage pro-inflammatory activation and multiple Toll-like receptors. Front Immunol.

[67] Lv K, Li Q, Zhang L (2019). Incorporation of small extracellular vesicles in sodium alginate hydrogel as a novel therapeutic strategy for myocardial infarction. Theranostics.

[68] Byts N, Makieieva O, Zhyvolozhnyi A (2023). Purification of Bacterial-Enriched Extracellular Vesicle Samples from Feces by Density Gradient Ultracentrifugation. Methods Mol Biol.

