# Supplementary material for: Enrichment of sweat-derived extracellular vesicles of human and bacterial origin for biomarker identification
Source: Nanotheranostics. 2024 Jan 1;8(1):48–63. doi: 10.7150/ntno.87822 (PMC10750121; doi:10.7150/ntno.87822)
Supplement: Supplementary file 1 — Supplementary figures and tables 3 and 7, table legends. [file ntnov08p0048s1.pdf]

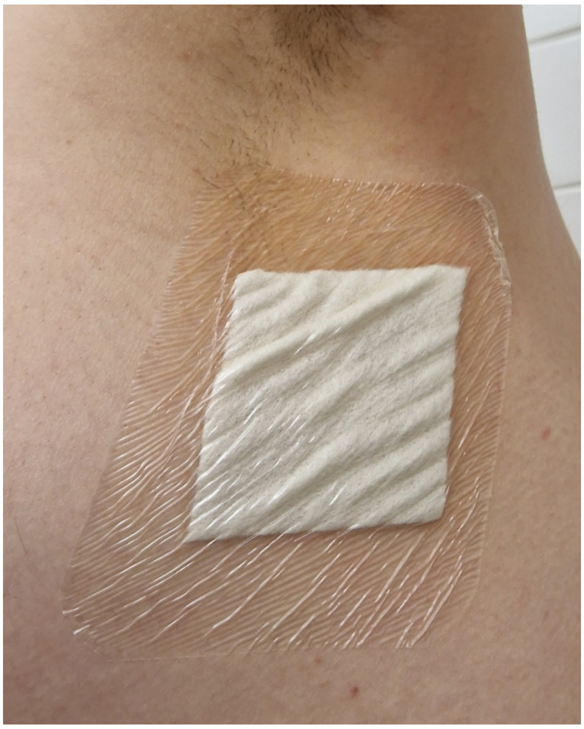

Suppl. Figure 1. Calcium alginate patch placed approximately below the armpit for sweat collection.

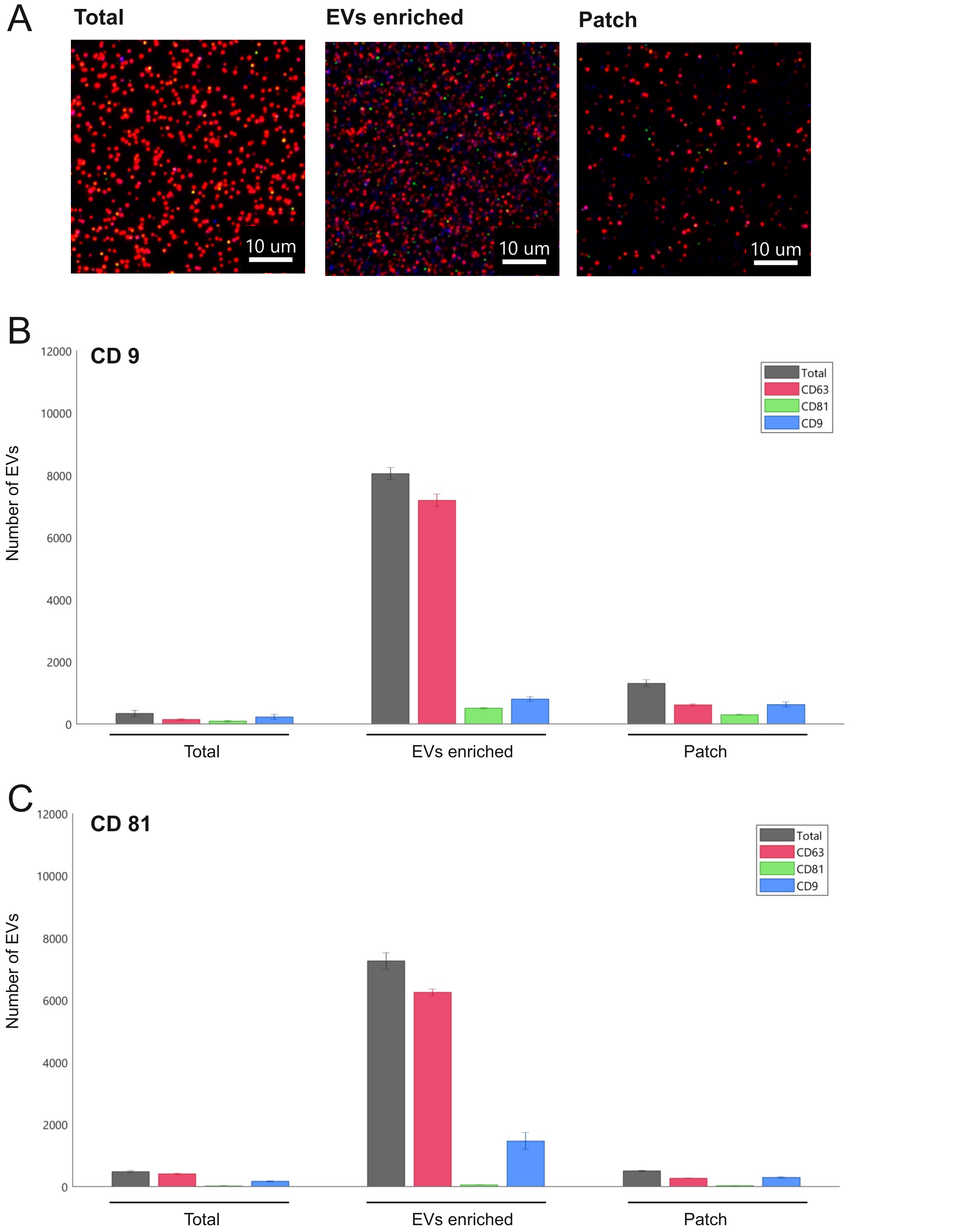

Suppl. Figure 2. Characterization of sweat EVs in total, EV enriched and patch with ExoView platform. (A) Representative images of individual slots on the chips. Colored dots represent single vesicles captured by CD63 antibodies anchored on corresponding slot. Dot's color depends on the fluorescently labelled detection antibodies (red for CD63, green for CD81, and blue for CD9). (B) Numbers of total, CD63-positive, CD81-positive and CD9-positive EVs captured on ExoVIew chip carrying CD9 antibody. (C)Numbers of total, CD63-positive, CD81-positive and CD9-positive EVs captured on ExoVIew chip carrying CD81 antibody.

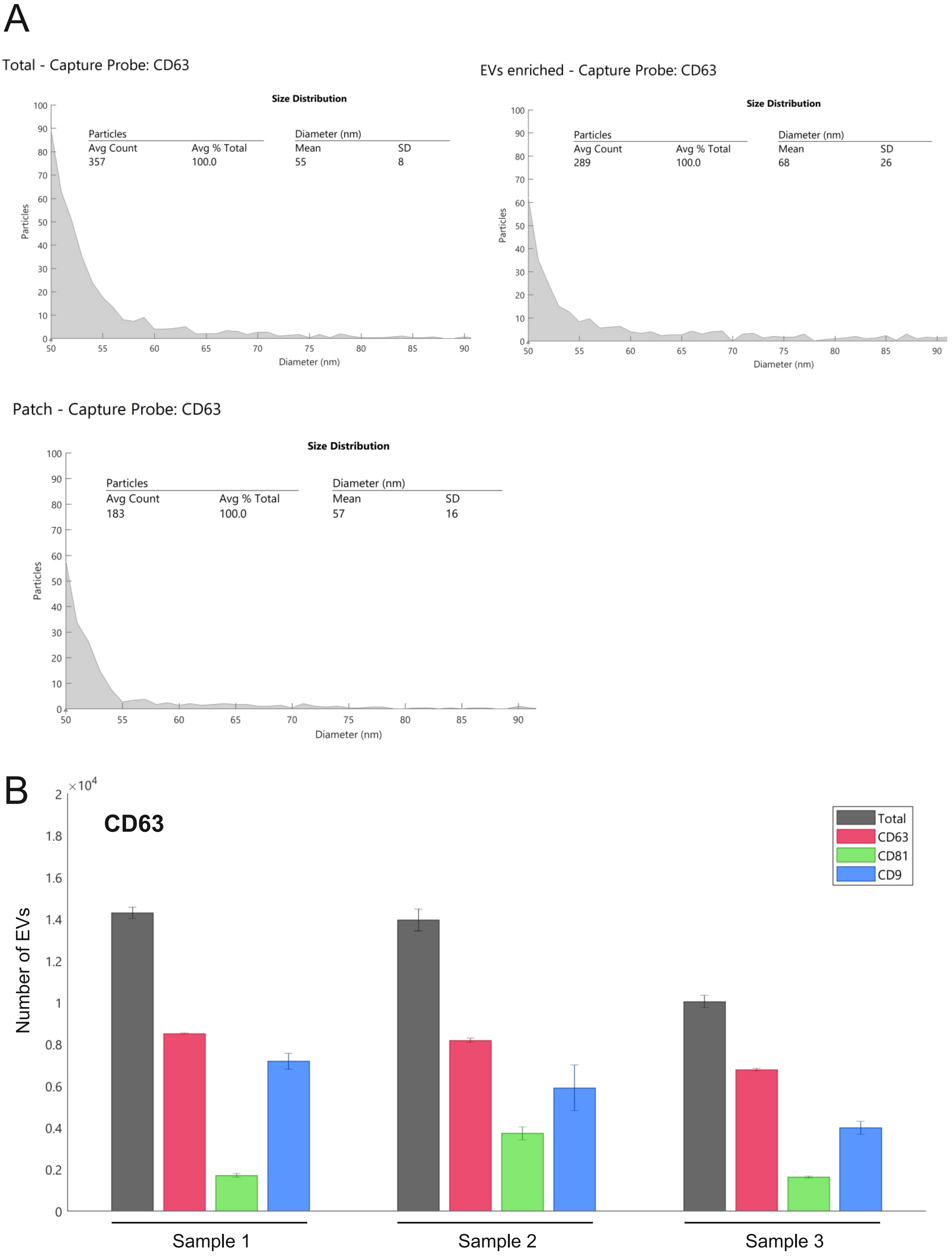

Suppl. Figure 3. (A) EV size distributions for total, EV enriched and patch sweat (ExoView analysis). (B) Characterization of EV enriched sweat samples from three volunteers with ExoView platform. See description for Figure 2B.

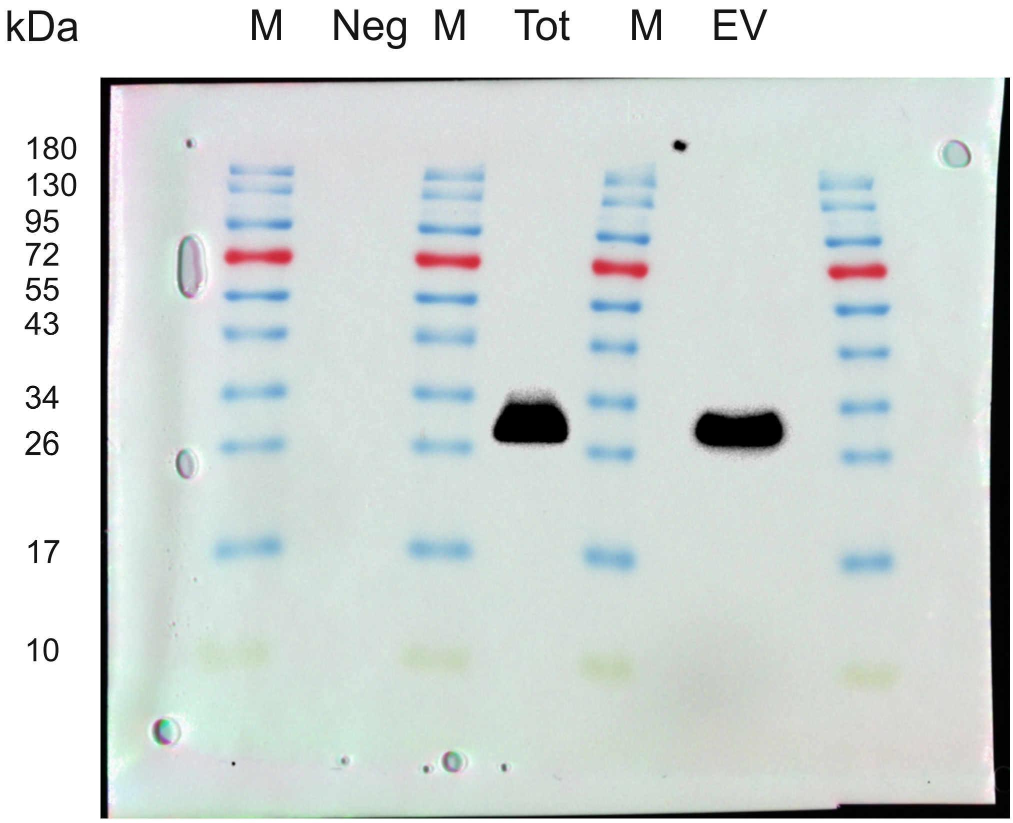

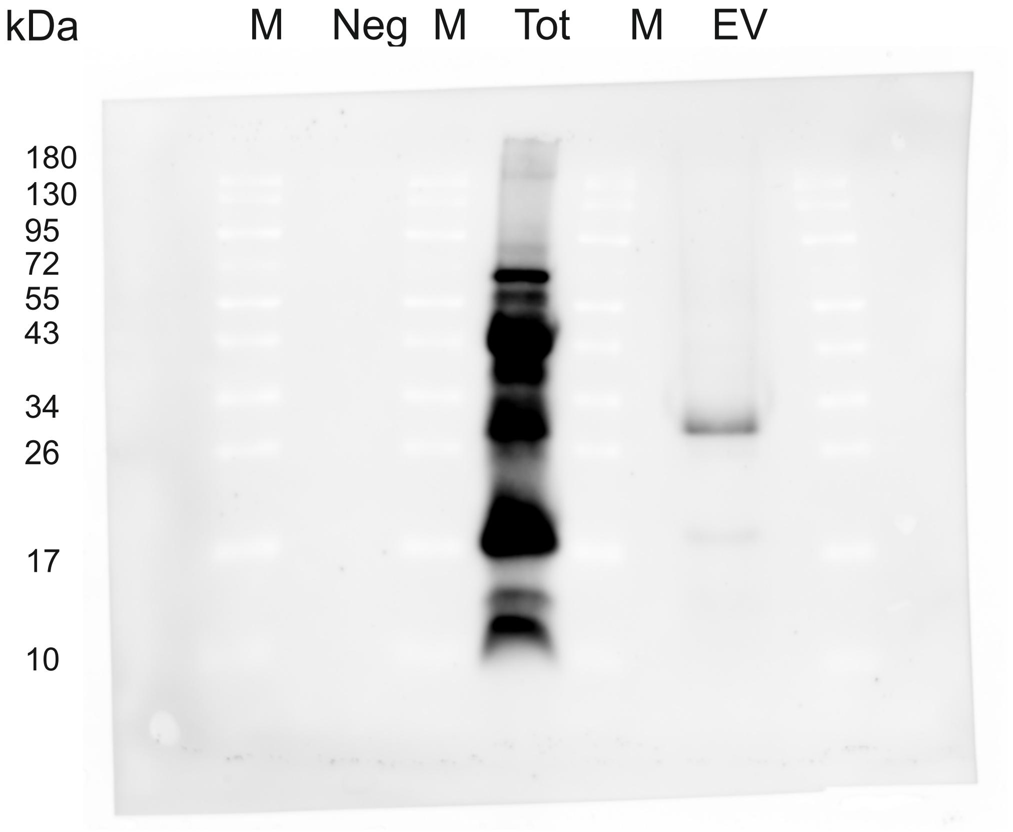

Suppl. Figure 4. Image of uncropped blot used for Figure 2. Western blot with anti-CD63 antibody (upper panel) and total protein stain of the same membrane (Azure Biosystems) (lower panel). M – marker (PageRuler Prestained Protein Ladder, Thermo Fisher, 26616). Neg – Negative control (PBS after washing of glove and processed the same as sweat sample), Tot – total, EV – EV enriched sample.

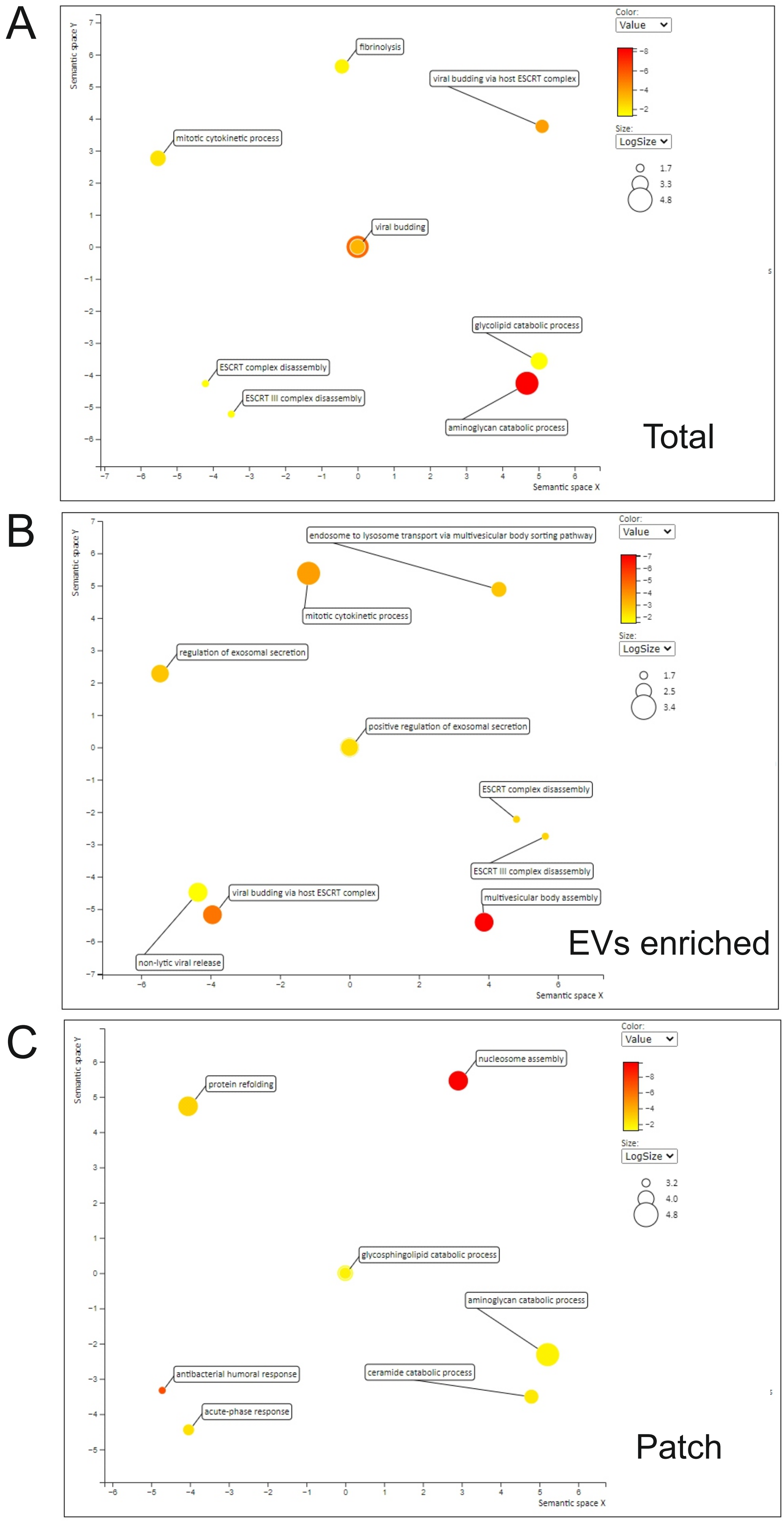

Suppl. Figure 5. Functional classification of sweat EV proteins by Gene Ontology (GO) annotations for biological processes for total (A), EV enriched (B) and patch (C) sweat. Only pathways showing at least 10-fold enrichment in PANTHER Overrepresentation Test (http://www.geneontology.org/) were used. Scatterplots were generated by a Web server REVIGO (http://revigo.irb.hr/). Bubble color indicates the Log10 p-value; bubble size indicates the log size of each group.

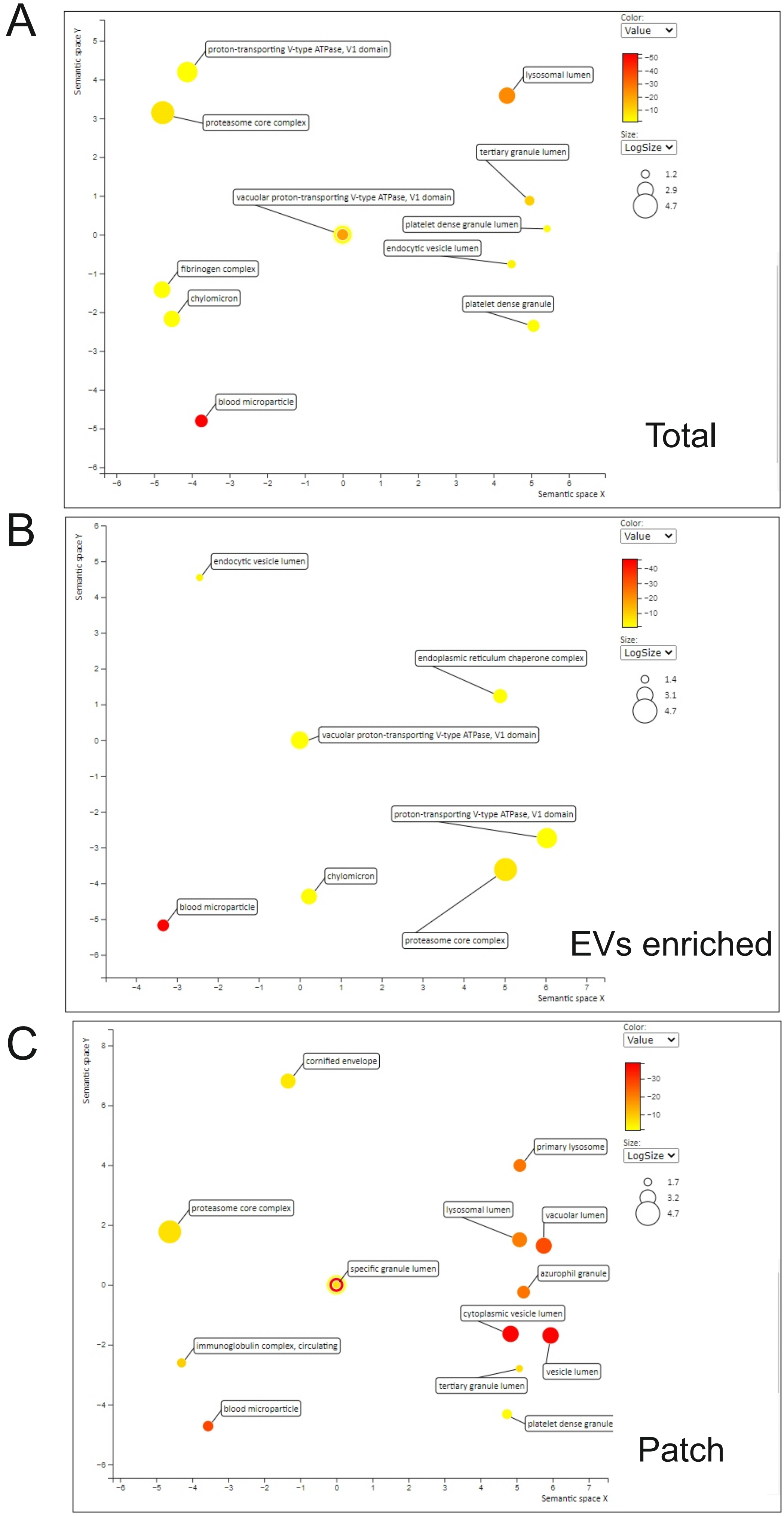

Suppl. Figure 6. Functional classification of sweat EV proteins by Gene Ontology (GO) annotations for cellular components for total (A), EV enriched (B) and patch (C) sweat. Only pathways showing at least 10-fold enrichment in PANTHER Overrepresentation Test (http://www.geneontology.org/) were used. Scatterplots were generated by a Web server REVIGO (http://revigo.irb.hr/). Bubble color indicates the Log10 p-value; bubble size indicates the log size of each group.

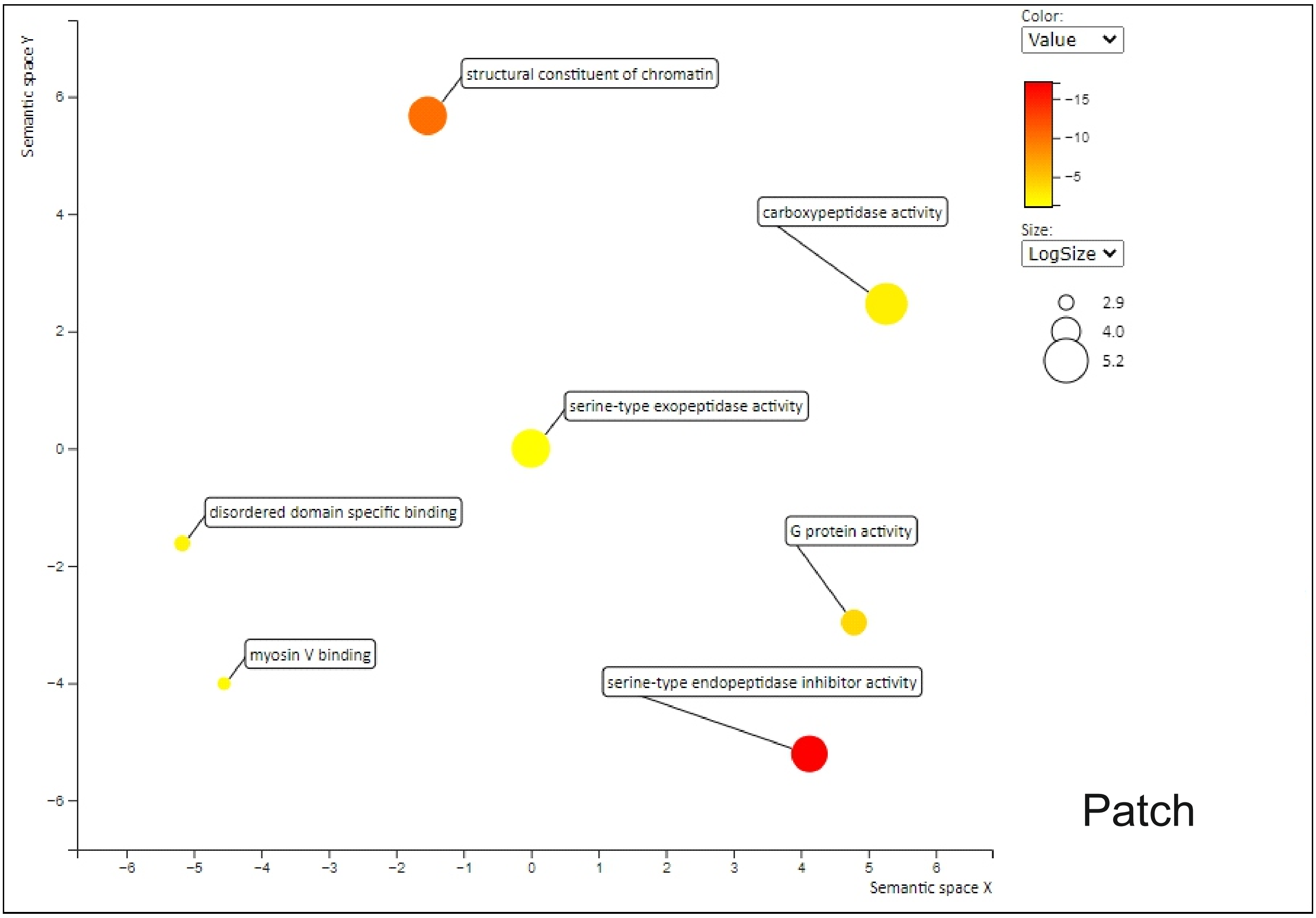

Suppl. Figure 7. Functional classification of sweat EV proteins by Gene Ontology (GO) annotations for molecular function for patch samples. Only pathways showing at least 10 fold enrichment in PANTHER Overrepresentation Test (http://www.geneontology.org/) were used. Scatterplots were generated by a Web server REVIGO (http://revigo.irb.hr/). Bubble color indicates the Log10 p-value; bubble size indicates the log size of each group.

A

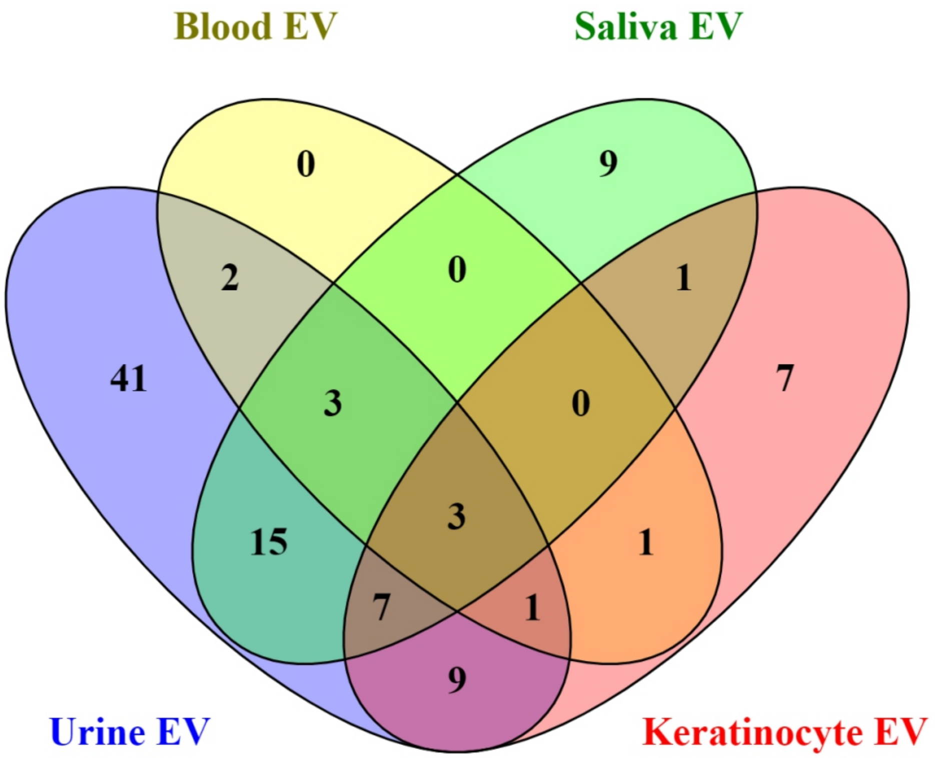

B

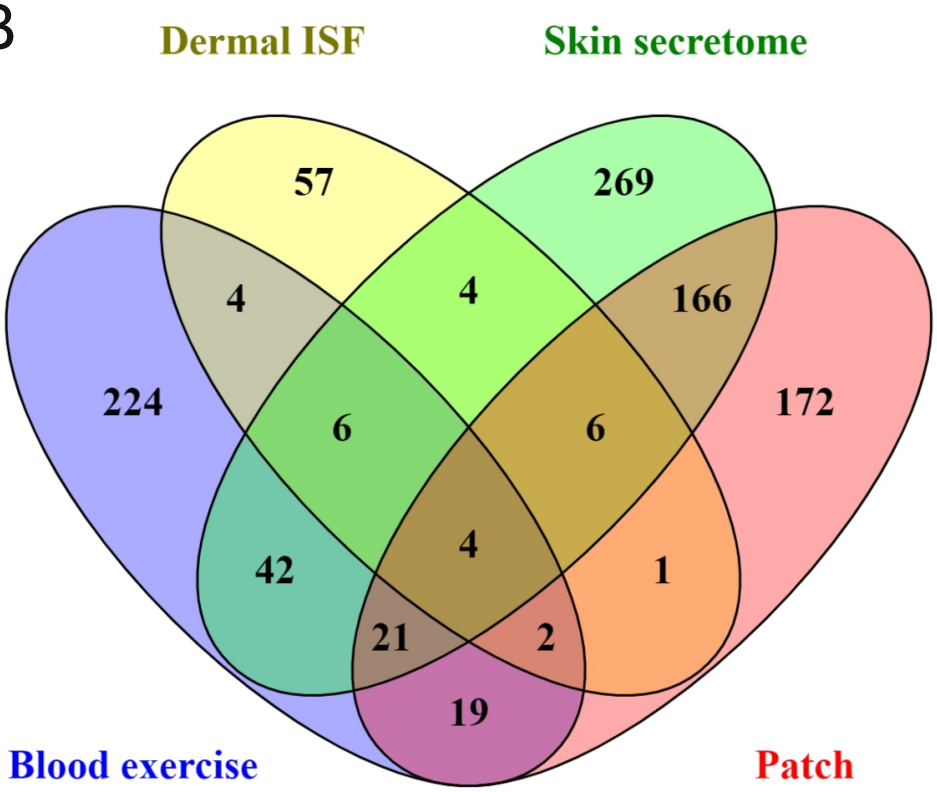

Suppl. Figure 8. (A) Comparison of proteins found in all EV-enriched sweat samples and bound by skin patches with ExoCarta data for human biofluids' and keratinocytes' Evs (http://www.exocarta.org). (B) Venn diagram comparing patch-bound sweat EV proteins identified in our study with skin washes (”skin secretome”), dermal interstitial fluid (”Dermal ISF”) (only proteins significantly higher expressed in ISF compared to plasma are analyzed), and blood EVs (only proteins significantly indued in plasma by exercise are analyzed) (”Blood exercise”) [45–47]. Actual protein lists used for comparisons are given in Suppl. Table 5.

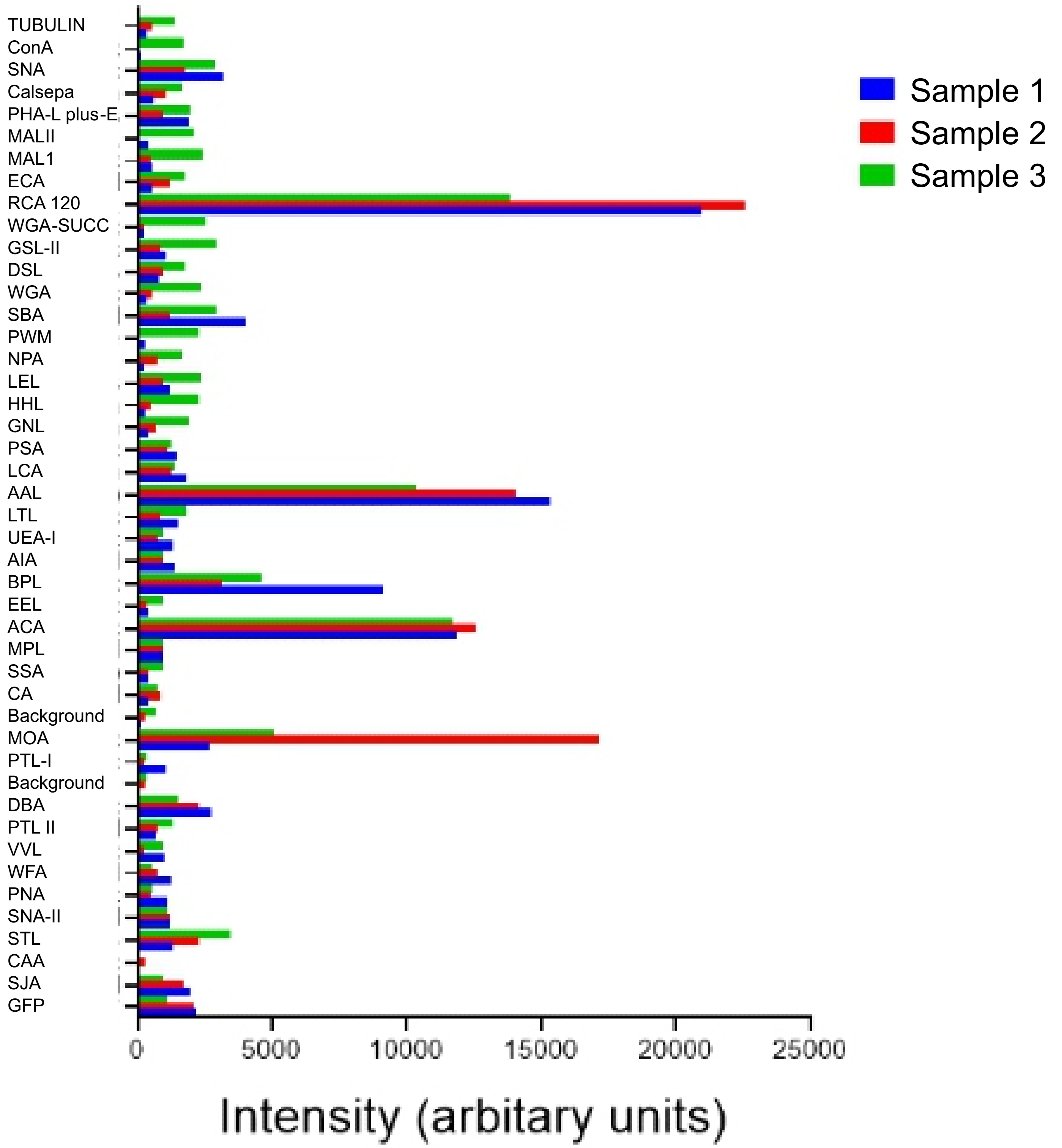

Suppl. Figure 9. Analysis of EV proteins glycosylation using lectin microarray. The mean intensities of bound label from 4 parallel arrays are shown after background subtraction. Three sweat EV samples listed to the right were analyzed.

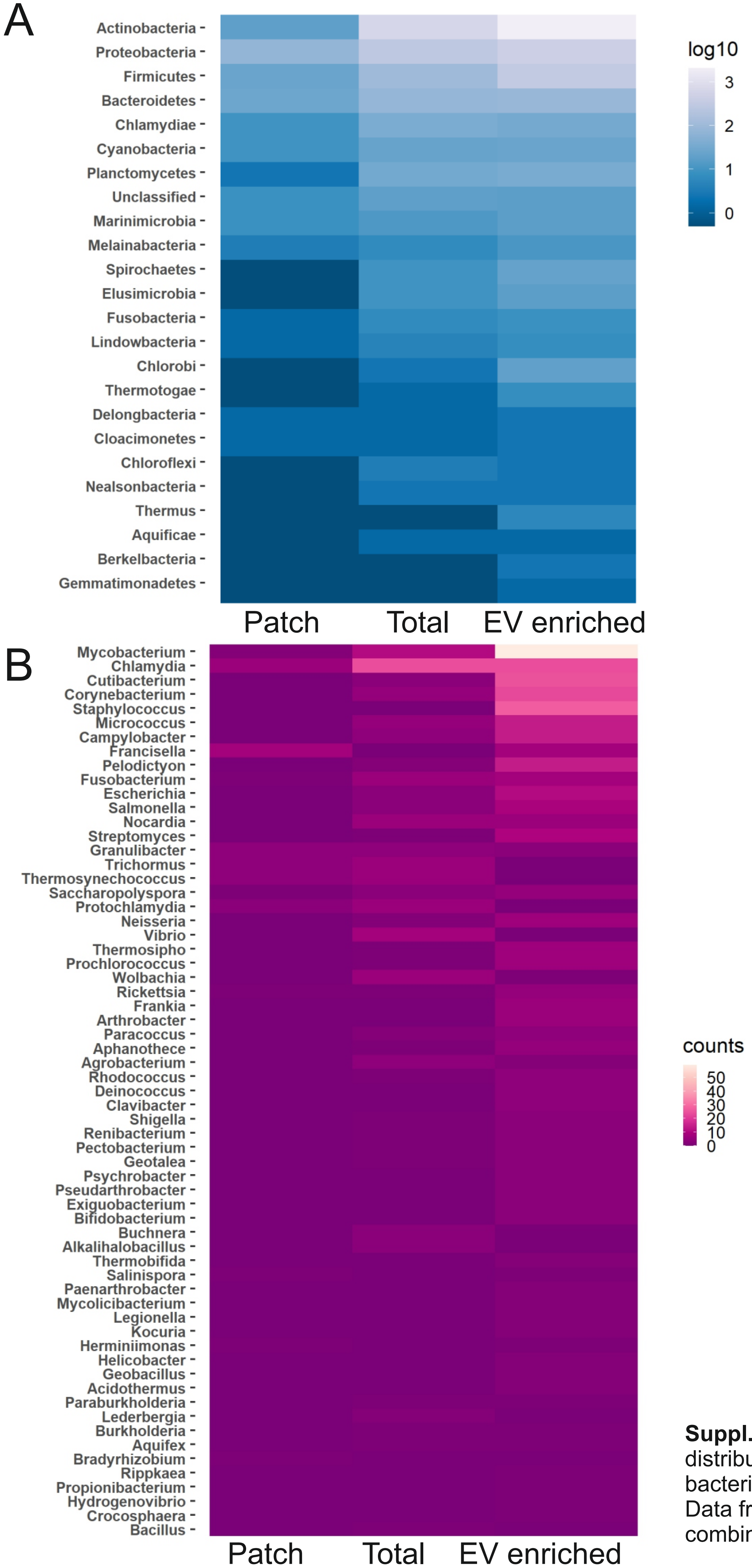

Suppl. Figure 10. Heatmaps showing distribution of EV proteins by different bacterial phylae (A) and genera (B). Data from all individual samples was combined for the analysis.

	EV enriched		Total		Patch	
	Percentage	SEM	Percentage	SEM	Percentage	SEM
DNA metabolism protein (PC00009)	**0,3875**	0,11259916	**0,55**	0,2	**0,625**	0,892428
RNA metabolism protein (PC00031)	**1,1375**	0,29730936	**1,2625**	0,381491	**1,0375**	0,936464
calcium-binding protein (PC00060)	**2,375**	0,28660575	**2,225**	0,361544	**3,35**	1,608904
cell adhesion molecule (PC00069)	**2,3875**	1,01198179	**2,4**	0,755929	**2,675**	1,807722
cell junction protein (PC00070)	**0,0875**	0,0834523	**0**	0	**0**	0
chaperone (PC00072)	**3,05**	0,27774603	**1,975**	0,565054	**5**	2,960212
chromatin/chromatin-binding protein (PC00077)	**1,6625**	0,77078346	**0,6625**	1,032248	**1,3125**	2,640583
cytoskeletal protein (PC00085)	**4,1625**	1,0676242	**2,9**	0,973946	**2,675**	2,799362
defense/immunity protein (PC00090)	**9,425**	2,11778186	**14,175**	2,711747	**8,5375**	3,88033
extracellular matrix protein (PC00102)	**1,2625**	0,23260942	**0,9875**	0,274838	**0,1375**	0,266927
gene-specific transcriptional regulator (PC00264)	**0,3625**	0,1685018	**0,3**	0,244949	**0,3625**	0,403334
intercellular signal molecule (PC00207)	**1,7375**	0,3335416	**1,925**	0,49208	**2,5625**	0,966492
membrane traffic protein (PC00150)	**3,8**	0,45981363	**3,1125**	0,659951	**3,5625**	1,863129
metabolite interconversion enzyme (PC00262)	**21,1125**	2,07601094	**22,9375**	1,197542	**14,1**	4,422023
protein modifying enzyme (PC00260)	**12,925**	0,86147714	**14,075**	1,372953	**14,3875**	1,327121
protein-binding activity modulator (PC00095)	**10,4125**	0,7698562	**9,1625**	0,924179	**14,6**	4,669965
scaffold/adaptor protein (PC00226)	**2,1625**	0,4838462	**1,675**	0,528475	**0,4**	0,632456
storage protein (PC00210)	**0,0125**	0,03535534	**0,025**	0,070711	**0**	0
structural protein (PC00211)	**0,0875**	0,06408699	**0**	0	**0**	0
transfer/carrier protein (PC00219)	**2,35**	0,70305456	**2,9125**	0,502671	**3,7625**	1,458901
translational protein (PC00263)	**0,6625**	0,30207615	**0,425**	0,243487	**1,35**	1,983503
transmembrane signal receptor (PC00197)	**2,1125**	0,4421942	**2,3**	0,778276	**2,0375**	1,469633
transporter (PC00227)	**2,9875**	0,6599513	**1,175**	0,78876	**0,25**	0,563154
viral or transposable element protein (PC00237)	**0,1375**	0,09161254	**0,05**	0,092582	**0**	0
Unclassified	**13,1625**	1,68687158	**12,8**	1,33095	**17,2375**	4,236554

	p values		
	EV enriched vs total	EV enriched vs patch	Total vs patc
DNA metabolism protein (PC00009)			
RNA metabolism protein (PC00031)			
calcium-binding protein (PC00060)			
cell adhesion molecule (PC00069)			
cell junction protein (PC00070)			
chaperone (PC00072)			
chromatin/chromatin-binding protein (PC00077)	0,0022		0,0481
cytoskeletal protein (PC00085)			
defense/immunity protein (PC00090)			
extracellular matrix protein (PC00102)	0,0026		0,0361
gene-specific transcriptional regulator (PC00264)		0,0001	0,003
intercellular signal molecule (PC00207)			
membrane traffic protein (PC00150)			
metabolite interconversion enzyme (PC00262)	0,0036		
protein modifying enzyme (PC00260)		0,0213	0,0013
protein-binding activity modulator (PC00095)			
scaffold/adaptor protein (PC00226)	0,0051		0,0452
storage protein (PC00210)		0,0003	0,0049
structural protein (PC00211)			
transfer/carrier protein (PC00219)			
translational protein (PC00263)	0,0412		
transmembrane signal receptor (PC00197)			
transporter (PC00227)			
viral or transposable element protein (PC00237)	0,0022	0,0001	0,0003
Unclassified			

Suppl. Table 3. All molecular classes identified in samples and statistical analysis results.

	Lectin
GFP	GFP
GalNAc > Gal	SJA
GalNAc	CAA
[GlcNAcβ(1,4)]2-4	STL
GalNAc = Lac > Gal	SNA-II
Galβ(1,3)GalNAc	PNA
GalNAc >> Lac > Gal	WFA
GalNAc	VVL
α-/β-linked GalNAc, Gal	PTL II
Terminal α-GalNAc	DBA
Gal, α-GalNAc	PTL-I
Galα(1,3)Gal or Galα(1,3)Galβ(1,4)GlcNAc	MOA
Lac > GalNAc > Gal	CA
GalNAc (Tn antigen)	SSA
Galβ(1,3)GalNAc > Gal	MPL
GalNAc, Galβ(1,3)GalNAc	ACA
α -Gal(1->3)(α-Fuc(1->2)-βGal(1->3)/4)-β-GlcNAc	EEL
Galβ(1,3)GalNAc, GalNAc	BPL
Galβ(1,3)GalNAc, α-Gal	AIA
α--Fuc	UEA-I
α-Fuc	LTL
Fucα(1,6)GlcNAc (core Fuc)	AAL
Fucα(1,6)GlcNAc, α-Man	LCA
α-Man, α-Glc	PSA
Manα(1,3)Man	GNL
Manα(1,3)Man or Manα(1,6)Man	HHL
GlcNAcβ(1,4)GlcNAc oligomers	LEL
α-Man	NPA
β(1,4)-linked (GlcNAc)n, N-acetyllactosamine	PWM
α-/β-GalNAc, Gal	SBA
(GlcNAcβ4)n, NeuAc	WGA
(GlcNacβ4)n, tri- and tetraantennary N-glycans	DSL
Agalactosylated tri/tetraantennary glycans, GlcNAc	GSL-II
[GlcNAcβ(1,4)]3 > [GlcNAcβ(1,4)]2 > GlcNAc >> Neu5Ac	WGA-SUCC
Galβ(1-4)GlcNAc-R	RCA 120
Galβ1-4GlcNAC, LacNAc > Lac > GalNAc, Gal	ECA
(Siaα(2,3))Galβ(1,4)GlcNAc	MAL1
Siaα(2,3)-Gal β1-4 GlcNAc β-Man-R	MALII
Gal, Complex triantennary N-linked glycans	PHA-L plus E
Man, High Man	Calsepa
Neu5Acα(2-6)Gal or Neu5Ac(2-6)GalNAc	SNA
α-Man > α-Glc > α-GlcNAc	ConA
Anti-Tubulin	ANTI-TUBULIN

Suppl. Table 7. The full list of lectins assayed and their sugar specificities.

Supplementary Tables.

Suppl. Table 1. PEAKS search results for UniProt SWISS-PROT and TrEMBL sequence databases.

Suppl. Table 2. List of all human proteins identified in total, EVs enriched and patch sweat samples.

Suppl. Table 3. List of all molecular classes (with GO annotations) identified in total, EVs enriched and patch sweat samples, and statistical analysis results.

Suppl. Table 4. All GO pathways ("biological processes", "cellular components", "molecular function") identified in sweat samples, and data of overrepresentation analysis.

Suppl. Table 5. Protein lists used in comparisons shown in Figure 4A, B, D [35,37,47–49].

Suppl. Table 6. Comparison of "core" EV proteins with previously reported EV markers from human biofluids and keratinocytes (http://www.exocarta.org).

Suppl. Table 7. The full list of lectins assayed and their sugar specificities.

Suppl. Table 8. List of all bacterial proteins identified in total, EVs enriched and patch sweat samples.

Suppl. Table 9. Distribution of bacterial proteins identified in individual samples by phylae.
